# Transition Metal‐Based Materials for Electrochemical and Photoelectrochemical Carbon‐Free Nitrogen Cycling as H‐Carrier

**DOI:** 10.1002/EXP.20240245

**Published:** 2025-10-02

**Authors:** Yuankai Li, Qian Lei, Won Tae Hong, Xinghui Liu, Chenyang Xue, Jung Kyu Kim

**Affiliations:** ^1^ School of Chemical Engineering Sungkyunkwan University (SKKU) Suwon Republic of Korea; ^2^ National Key Laboratory of Aerospace Chemical Power Hubei Institute of Aerospace, Chemotechnology Xiangyang Hubei China; ^3^ Pen‐Tung Sah Institute of Micro‐Nano Science and Technology Xiamen University Xiamen China; ^4^ SKKU Advanced Institute of Nanotechnology (SAINT) Sungkyunkwan University (SKKU) Suwon Republic of Korea

**Keywords:** electrochemical, green hydrogen, nitrogen cycling, photoelectrochemical, transition metal‐based materials

## Abstract

Ammonia, as a carbon‐free nitrogen‐based hydrogen carrier, has attracted significant interest in addressing the approaching energy model innovation in light of its high hydrogen content, low cost, ease of storage, and transport. However, the additional energy consumption and environmental pollution caused by the traditional Haber–Bosch ammonia production and thermal ammonia catalytic cracking process enforce the exploration of clean and renewable ammonia cycling approaches. Electrochemical (EC) and photoelectrochemical (PEC) ammonia synthesis and oxidation for hydrogen generation have shown great potential for achieving an eco‐friendly and sustainable green hydrogen economy. Exploring low‐cost, highly active, and stable catalysts is pivotal for both EC and PEC systems to achieve efficient ammonia conversion properties. Transition metal‐based catalysts (TMCs) have shown significant potential in EC and PEC catalytic systems because of their high catalytic activity, low cost, and excellent stability. We summarize the recent advanced progress of TMCs applied to EC and PEC ammonia synthesis and decomposition to hydrogen generation. Moreover, we discuss the challenges and perspectives on exploring transition metal‐based materials in EC and PEC ammonia conversion. This review offers guidance for developing carbon‐free nitrogen cycling as a hydrogen carrier.

## Graphical abstract

This review summarizes the recent advanced progress of transition metal‐based catalysts used for electrochemical and photoelectrochemical ammonia synthesis and decomposition for hydrogen generation. The conclusion encapsulates the current challenges and prospects for the exploration of transition metal‐based materials in green ammonia conversion.

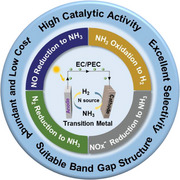

## 1 Introduction

Over the past few decades, along with the rapid development of industrial technology and the continuous rise in the global population, human reliance on fossil fuels for energy, including predominantly coal, oil, and natural gas, is approaching its availability limits [1]. Moreover, the corresponding environmental issues and geopolitical instability caused by the consumption of fossil fuels are becoming increasingly severe. Therefore, exploring clean, renewable, low‐cost, and more evenly distributed energy resources is crucial for sustainable and stable development [2]. Hydrogen is considered the most sustainable and compelling candidate for a clean energy carrier owing to its high energy density (142 MJ kg^−1^), carbon‐free emissions, and sustainability [3]. However, under harsh conditions, hydrogen production is currently suffering from the carbon dioxide emissions and unsustainability associated with traditional steam reforming methods. In addition, the low liquefication temperature of hydrogen also limits its storage and transportation. Therefore, green hydrogen generation and cost‐conducive and safe hydrogen storage are particularly crucial in fully addressing the hydrogen economy concept [4, 5]. Recently, nitrogen‐based energy carriers for energy storage have shown the potential to discourage the utilization of fossil fuels, which could beneficially address the issue of greenhouse gas emissions from CO_2_ [6, 7]. Ammonia (NH_3_), a cost‐effective nitrogen‐based hydrogen carrier, has proven to be a promising platform molecule for the emerging renewable and green hydrogen economy [8, 9].

NH_3_ is primarily used in modern agriculture, while the global ammonia production exceeds 170 million tons annually [10]. Since the early 1900s, when the Haber–Bosch process was invented, this thermo‐catalytic process for synthesizing NH_3_ from hydrogen (H_2_) and nitrogen (N_2_) has been unparalleled in the industrial production of NH_3_, following continuous optimization, holding an unbeatable monopoly position [11]. Nevertheless, the corresponding energy wastage and environmental issues associated with this carbon‐intensive ammonia production process cannot be ignored. The Haber–Bosch process requires high temperature and high pressure (typically 700 K, 100 bar) and also relies entirely on hydrogen derived from the reforming of fossil hydrocarbons, which results in significant emissions of carbon dioxide (exceeding 300 million tons) [12]. Therefore, developing highly efficient, environmentally friendly, cost‐effective, renewable energy‐driven ammonia production processes is crucial. Besides its use in fertilizer production, NH_3_, as a carbon‐free material, is also considered an excellent hydrogen storage medium (17.7wt%) that can power hydrogen fuel equipment. Compared with hydrogen, NH_3_ can be easily liquefied (8 bar) and provides a high volumetric energy density of 10.5 MJ L^−1^, which can significantly reduce the cost of storage and transportation compared to H_2_ (700 bar) [12]. Therefore, NH_3_ is a good candidate for energy storage, while efficient NH_3_ conversion to H_2_ is crucial for the overall hydrogen economy. Moreover, H_2_ production from additional NH_3_ sources, such as wastewater, could benefit environmental improvement and sustainable energy development [13].

**FIGURE 1:**
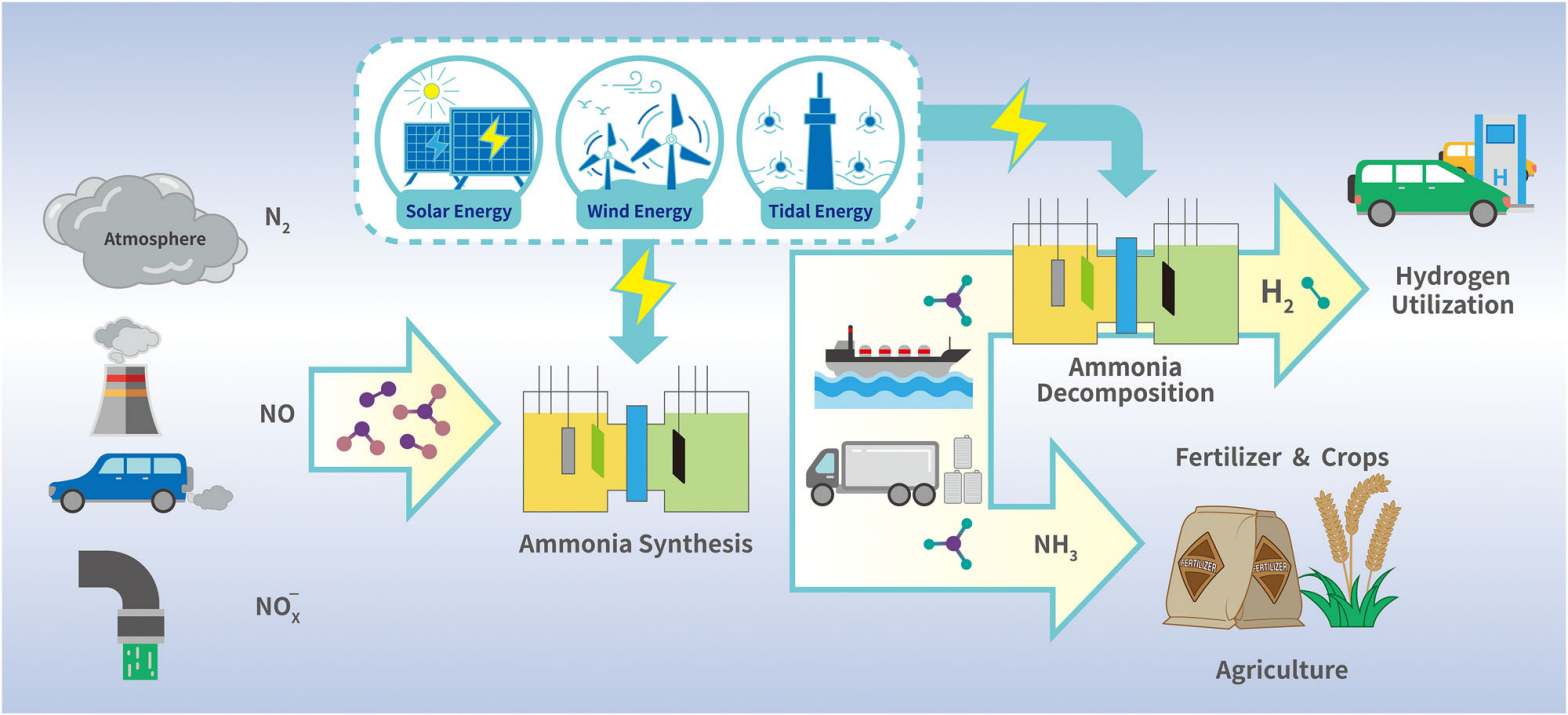
Schematic illustration of EC/PEC ammonia production and utilization as H‐carrier.

In recent years, electrochemical (EC) and photoelectrochemical (PEC) catalytic processes for NH_3_ synthesis and decomposition have attracted increasing interest, benefiting from their potential advantages in terms of cost, environmental friendliness, and safety [4, 14, 15, 16, 17]. The artificial EC synthesis of NH_3_ can be coupled with renewable and clean electricity, directly converting the energy contained in renewable sources into chemical energy, thereby simplifying the entire conversion process and improving efficiency. Compared to the Haber–Bosch process, electrocatalytic NH_3_ synthesis can be operated under milder conditions without CO_2_ emission, making it more environmentally friendly and cost‐effective. For the EC NH_3_ synthesis, lots of research utilizes N_2_ as the nitrogen source and H_2_O as the hydrogen source [18]. At the anode part, an oxygen evolution reaction (OER) occurs to produce O_2_, while at the cathode part, a nitrogen reduction reaction (NRR) takes place to synthesize NH_3_. However, suffering from the high bonding energy of the N_2_ molecule (N≡N 941 kJ mol^−1^), the efficiency of NRR to produce NH_3_ falls short of expectations [19]. Besides N_2_ molecules, environmentally harmful reactive nitrogen sources $\left(\mathrm{NO},{\mathrm{NO}}_{x}^{-}\right)$ are considered attractive precursors for achieving effective NH_3_ production. Moreover, the development of EC/PEC NO reduction reaction (NORR) and ${\mathrm{NO}}_{x}^{-}$ reduction reaction (NtrRR) to NH_3_ holds significant importance for water purification and resource recovery [19]. On the other hand, releasing the hydrogen energy stored in NH_3_ efficiently is also challenging for developing the hydrogen economy. The release of H_2_ from NH_3_ vessel follows the Equation (1):

(1) $$NH_{3}\to \frac{1}{2}N_{2}+\frac{3}{2}H_{2}$$

where the decomposition process is mildly endothermic under **△**_rxn_ *H*° = 45.9 kJ mol^−1^, however, to achieve the desired decomposition rate, a high temperature over 400°C is required to overcome the kinetic barriers and thermodynamic limitations [16]. The harsh conditions raise concerns about the economic feasibility and safety issues of the reaction. Recently, electrocatalytic NH_3_ decomposition, as an alternative approach to high‐temperature thermal decomposition, has shown tremendous potential in achieving efficient conversion of NH_3_ owing to its mild oxidation condition and zero CO_2_ emission. In this context, ammonia synthesis and decomposition to hydrogen generation triggered by renewable electric energy show significant potential for the development of a carbon‐free energy economy, as outlined in Figure 1. First, different N sources, including nitrogen gas, nitric oxide, or nitrate, are applied for EC/PEC ammonia synthesis, which achieves energy storage. Then, benefiting from the well‐developed worldwide NH_3_ storage and transportation network, the produced ammonia can be transferred to the demanded sites for further EC/PEC decomposition to hydrogen generation, which is considered an economically friendly, safe, and sustainable energy system.

**FIGURE 2:**
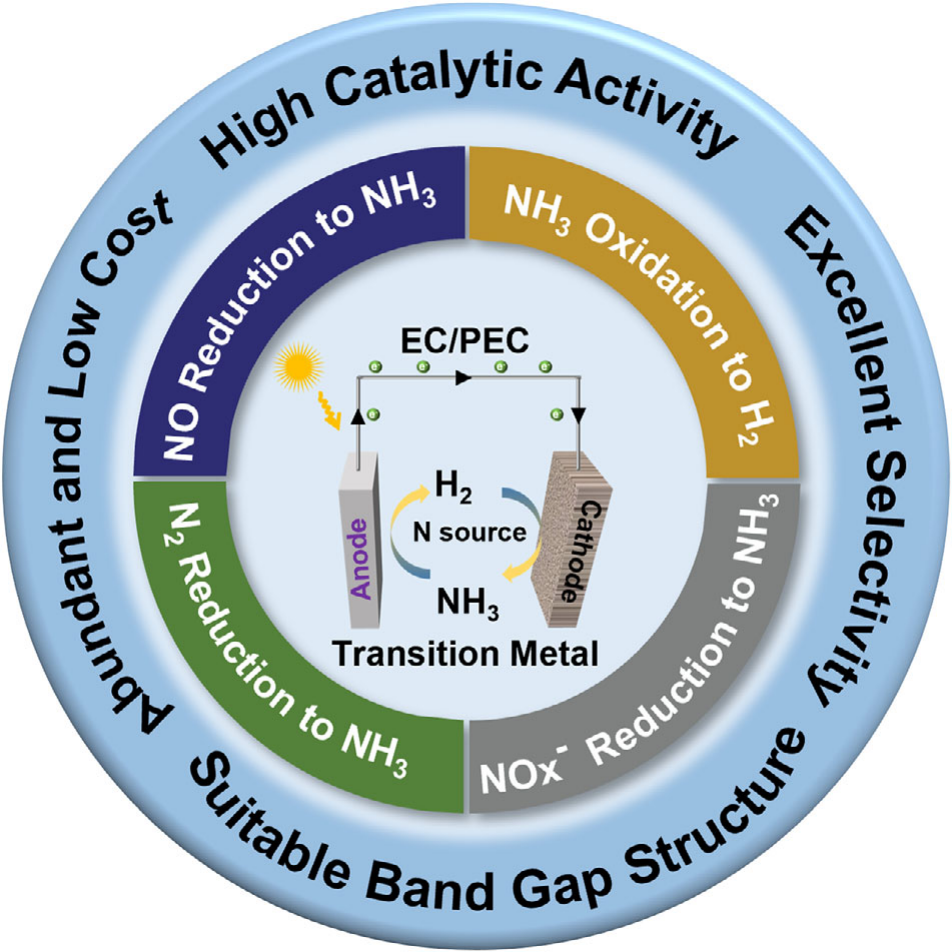
Schematic illustration of transition metal‐based catalyst for EC/PEC ammonia synthesis and decomposition.

Overall, catalysts are the most crucial factor in evaluating system properties for PEC (EC) catalytic systems for NH_3_ synthesis or NH_3_ decomposition to H_2_. Noble metals such as Ru, Pt, and Au have been demonstrated to be highly efficient catalysts for NH_3_ cycling. However, the inherent nature of scarcity seriously limits their large‐scale commercial applications [19]. Transition metal‐based catalysts (TMCs) with tunable features have been widely used in EC and PEC energy conversion systems due to their high catalytic activity, cost‐effectiveness, and stability [20, 21]. In light of this, many excellent corresponding sub‐research studies have been published recently, including EC/PEC NH_3_ synthesis and NH_3_ fuel cells for H_2_ generation [14, 16, 22]. Given the vast potential of carbon‐free clean energy development in the future green hydrogen economy, it is imperative to provide an overview of the current status and challenges of catalysts for the EC/PEC‐synthesis and decomposition of NH_3_. Herein, we summarize the recent reports on TMCs applied for EC/PEC carbon‐free hydrogen carrier ammonia cycling (Figure 2). First, each half‐reaction (NH_3_ synthesis and NH_3_ decomposition to H_2_) by using the EC/PEC strategy is elucidated. Then, the reported TMs applied for the EC/PEC NH_3_ synthesis and NH_3_ decomposition within 5 years are summarized. Finally, we summarize the challenges and prospects faced by transition metal‐based materials in NH_3_ synthesis and decomposition, which will benefit the rational design of highly efficient catalysts in the EC/PEC NH_3_ cycling system.

## 2 Advanced TMCs for NH_3_ Synthesis

**FIGURE 3:**
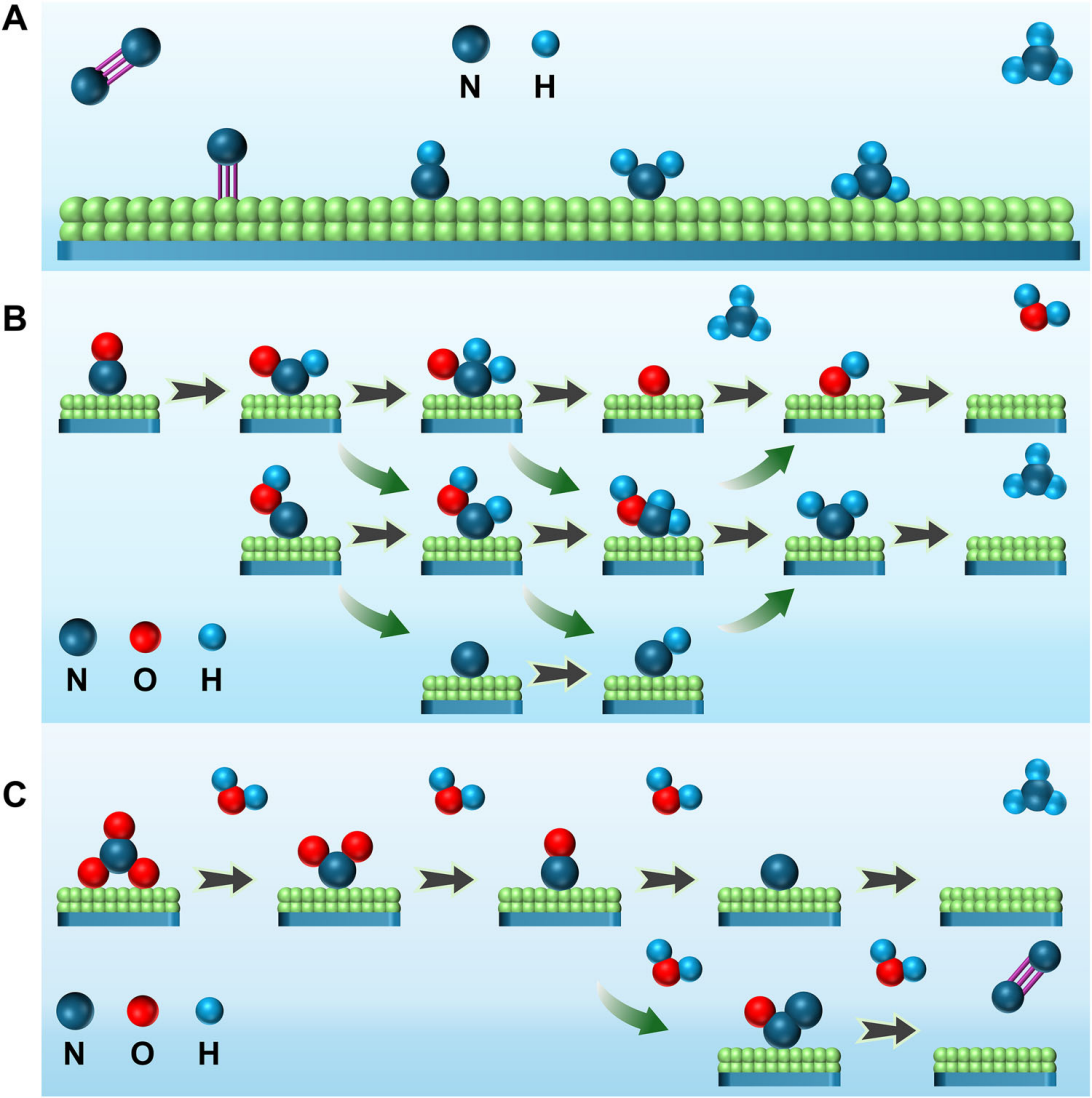
Reaction mechanisms illustration for EC ammonia synthesis. (A) EC NRR. Reproduced with permission [28]. Copyright 2019, American Chemical Society. (B) EC NORR [30]. Copyright 2024, Royal Society of Chemistry. (C) EC NtrRR. Reproduced with permission [19]. Copyright 2023, Elsevier.

EC and PEC conversion processes can actuate chemical reactions under normal pressure and temperature (NPT), converting N_2_, NO, and NO*_x_* into NH_3_ by using renewable electricity as an energy source [23, 24, 25, 26]. Compared with the Haber–Bosch process, EC and PEC processes can reduce energy consumption and eliminate CO_2_ emissions caused by steam‐reforming fossil fuels. Moreover, the EC and PEC processes offer huge potential for on‐site/‐demand and decentralized NH_3_ synthesis, which is beneficial for reducing the transportation cost of NH_3_ [19]. The EC NH_3_ synthesis can be retrospected to the 1960s when Professor Van Tamelen achieved the NRR to NH_3_ in the glyme electrolyte containing titanium tetraisopropoxide, tetrabutylammonium chloride, naphthalene, and aluminum isopropoxide, while the aluminum and nichrome served as anode and cathode, respectively [27]. Nevertheless, it was not until recent years that prolific progress was made when advanced materials with tunable composition, size, and morphology were developed as potential NRR electrocatalysts. The electrocatalysis of NRR to NH_3_ typically involves N_2_ adsorption, N≡N bond breaking and hydrogenation, and NH_3_ desorption (Figure 3A), where the breaking of N≡N requires high energy [28]. On the other hand, the electrosynthesis of NH_3_ from NRR suffers from the poor activity and low selectivity of the catalysts, where the hydrogen evolution reaction (HER) usually dominates in aqueous electrolysis systems, leading to poor Faradaic efficiency (FE) for NRR [29]. Electrocatalytic NORR is another approach for NH_3_ synthesis, while NO has lower bond energy (N═O, 204 kJ mol^−1^) compared to N_2_, which makes NORR more favorable to thermodynamics than NRR. The EC NORR abides by the associative or dissociative mechanism (Figure 3B) [30]. For the associative route, NO molecules are first hydrogenated into H*_x_* NOH*_y_* intermediates, then NH_3_ is released by the following stepwise hydrogenation. For the dissociative route, the NO molecules are first broken up and absorbed on the surface of the catalyst, followed by hydrogenation. Due to the 5e^−^ conversion of NORR to NH_3_, the crucial point for highly efficient NORR would be suppressing the competitiveness HER and enhancing the electron utilization. NO_3_^−^ is another N‐species widely used in global N‐economic; nevertheless, the surge of NO_3_^−^/NO_2_^−^ (NO*_x_* ^−^) pollutants resulting from human activities poses a major threat to the water environment and human health, and even leads to global N‐cycle imbalances. Electrocatalytic reduction of harmful NO*_x_* ^−^ (NtrRR) to value‐added NH_3_ can be a promising strategy for sewage clarification and carbon‐free energy conversion. As shown in Figure 3C, for the reaction pathway of direct electrocatalytic NO_3_^−^ (<1 mol L^−1^), the adsorbed NO_3_^−^_(ads)_ can be reduced to NO_2_^−^_(ads)_ through the transfer of two e^−^ and two H^+^. After that, the NO_2_^−^_(ads)_ can be further reduced to NO_(ads)_. Specifically, if the NO_(ads)_ reacts with the dissolved NO, the NtrRR would lead to the N_2_ generation, if the NO_(ads)_ is reduced to HNO_(ads)_, the NtrRR pathway would lead to the NH_3_ generation [19]. Given the complexity of reaction pathways and intermediates, improving the selectivity of NH_3_ synthesis is a significant challenge for NtrRR. Compared with EC NH_3_ synthesis, PEC NH_3_ production can directly use clean and renewable solar energy for nitrogen fixation. Moreover, the photoelectric synergies can significantly improve the separation efficiency of photogenerated electron/hole pairs, resulting in highly efficient utilization of solar energy and higher NH_3_ productivity. For the PEC NH_3_ synthesis approach, the p‐type semiconductor photocathode is the crucial point for the NH_3_ yield efficiency of the system, resulting from the light absorption and N‐source fixation mainly occurring on the surface of the photocathode. Hence, for the EC and PEC systems, developing highly efficient catalysts is critical for highly efficient NH_3_ production. In light of this, numerous efforts have been devoted to creating high‐performance catalysts, including noble metal‐based and non‐noble metal‐based catalysts.

Noble metal‐based catalysts, such as Ru, Au, Pd, and Ag, have demonstrated excellent properties for NH_3_ synthesis. Nevertheless, their intrinsic scarcity seriously limits their future development in practical N‐cycling applications. In addition, owing to their low‐cost, earth‐abundant properties and similar to the properties of nitrogenase in nature, which contain Fe, Mo, and Ni, non‐noble TMCs are considered promising candidates for ammonia synthesis [31, 32]. This section discusses the non‐noble TMCs for EC and PEC ammonia synthesis at length.

### 2.1 TMCs for EC NH_3_ Synthesis

#### 2.1.1 TMCs for EC NRR

**FIGURE 4:**
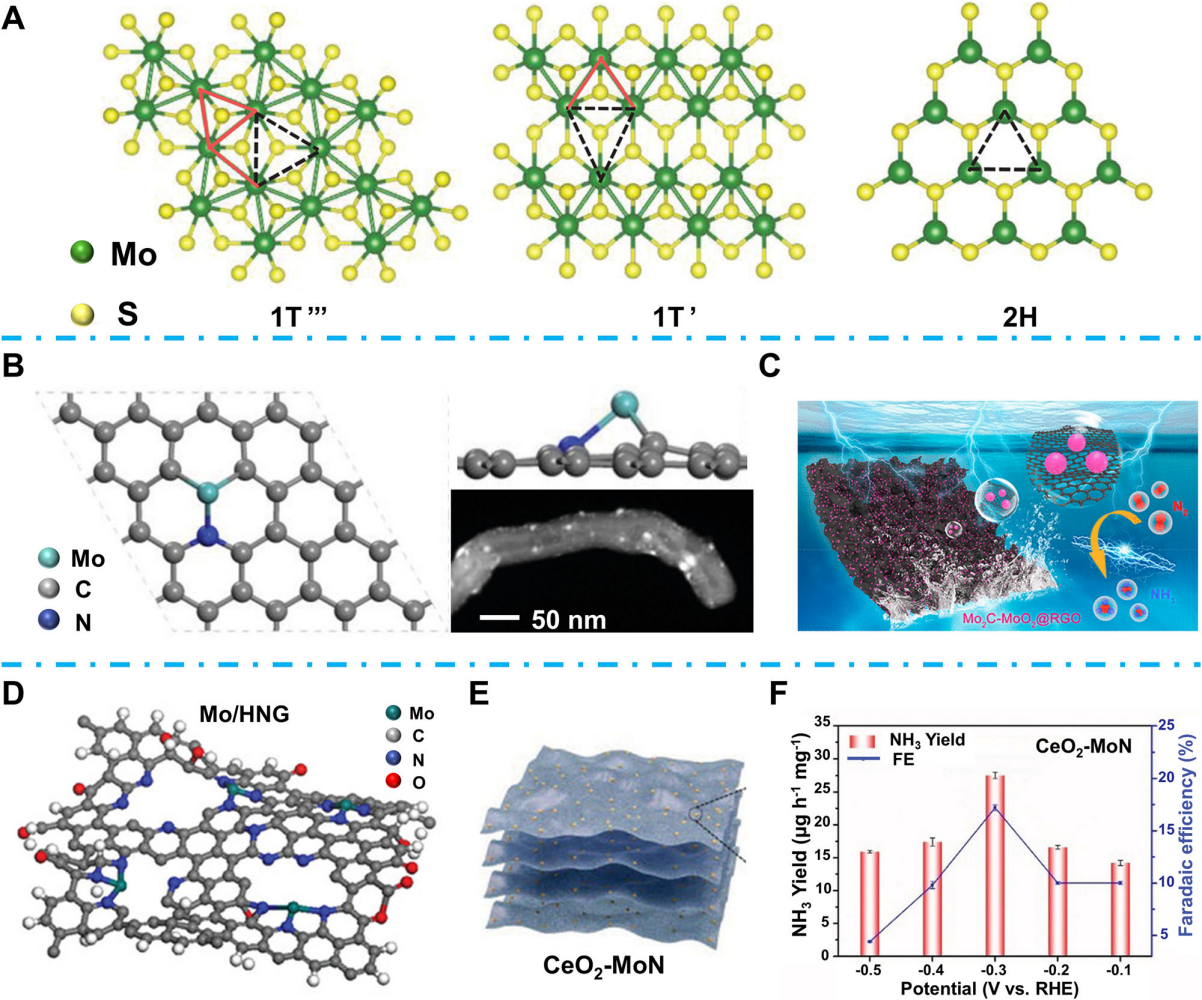
(A) Schematic of three different‐phases MoS_2_, green sphere for Mo atoms and yellow for S atoms. Reproduced with permission [43]. Copyright 2021, Wiley‐VCH. (B) Atomic structure of Mo_2_C (1 0 1). Reproduced with permission [45]. Copyright 2020, Wiley‐VCH. (C) Schematic illustration of Mo_2_C‐MoO_2_@RGO catalyst. Reproduced with permission [46]. Copyright 2022, American Chemical Society. (D) Schematic illustration of Mo/HNG catalyst. Reproduced with permission [47]. Copyright 2022, Wiley‐VCH. (E) Schematic illustration of CeO_2_/MoN catalyst, and (F) NH_3_ yield rate and FE of CeO_2_/MoN catalyst. Reproduced with permission [50]. Copyright 2024, Wiley‐VCH.

Inspired by natural nitrogenases, Mo and Fe‐based TMCs are widely studied in the EC NRR process and have demonstrated excellent properties in NH_3_ synthesis [33]. Most specifically, owing to the top position of N_2_ binding energy volcano diagram, many kinds of Mo‐based catalysts have been applied for EC NRR, including MoS_2_ [34], Mo*_x_* C [35], MoN [36], and single molybdenum atoms [37]. Considering the crucial function of Mo and S elements in the natural nitrogenases, Sun et al. first explored the activity of MoS_2_ in NRR. Combined with theoretical calculation and experimental verification, they demonstrated that MoS_2_ is an excellent candidate for EC NRR owing to the high active Mo ion at the edge of MoS_2_, while this groundbreaking research for MoS_2_ revealed excellent NH_3_ yield rate (8.08 × 10^−11^ mol s^−1^ cm^−1^) and high FE of 1.17% at −0.5 V_RHE_ [38]. After that, different strategies, including element doping and defect engineering, were applied to the MoS_2_ EC NRR optimization [39, 40, 41, 42]. Liu et al. proposed that the selectivity and activity of NRR are highly related to the concentration of S‐vacancy; their MoS_2_‐7H with optimized S‐vacancy (17.5%) shows excellent NH_3_ yield property (66.74 µg h**^−^** ^1^ mg**^−^** ^1^, −0.6 V_RHE_) and the FE for NH_3_ reaches 14.68% at −0.5 V_RHE_ [39]. Unlike most related research based on stable 2H phase MoS_2_, Lin et al. investigated the properties of metastable MoS_2_ (1T‴ and 1T′) in NRR [43]. As shown in Figure 4A, the Peierls distortion resulting from the altered symmetry of metastable 1T MoS_2_ leads to Mo−Mo clustering, which results in the different arrangements of Mo atoms named trimerization (1T‴) and zigzag chainlike clustering (1T′). This unique Mo−Mo interaction in metastable MoS_2_ can accelerate the electron transfer efficiency and facilitate the N_2_ activation. In particular, for the metastable MoS_2_, the localized electron state caused by the deformation of the Mo−S cluster can result in the upshift of D band of Mo. During the N_2_ fixation step of NRR, the upshift of Mo D band is favorable for the acceptance of lone‐pair electrons from nitrogen and electron transfer from Mo to N_2_ π antibonding orbital. As a result, the metastable MoS_2_ shows nine times NRR activity enhancement compared to the stable 2H phase. Li et al. proposed a S‐doped Mo single atom catalyst (SACs) which highly boosted the NRR FE to 28.9% at −0.2 V_RHE_, while the optimized NRR property is due to the modulated electron distribution of Mo atoms through S doping [37]. Mo_2_C is also considered a promising catalyst for NRR owing to the property of adsorbates of π orbitals [44]. Ma et al. reported an integrated synergy MoSAs‐Mo_2_C catalyst, as shown in Figure 4B [45]. To overcome the drawbacks of the unsatisfied adsorption of intermediates in the NRR process, a new mechanism to combine two catalysts is proposed to enhance the EC NRR property. They simultaneously assembled the Mo SAs and Mo_2_C nanoparticles on N‐doped C tubes and achieved four‐time enhancements compared to both single Mo and Mo_2_C. Wan et al. proposed another bicatalytic site synergy by combining the Mo carbide and Mo oxide, Mo_2_C‐MoO_2_ (Figure 4C) [46]. This research indicates that the inappropriate H adsorption of MoO_2_ could efficiently suppress the competitive HER and enhance the selectivity and FE of NRR to NH_3_ generation. Specifically, they fabricated the heterostructured Mo_2_C‐MoO_2_ and regulated the size to quantum dots (QDs), which can expose rich active sites and promote reaction kinetics. Moreover, the RGO substrate further enhanced the interfacial charge transfer, which resulted in an excellent NH_3_ yield rate of ≈14 µg h**^−^** ^1^ mg**^−^** ^1^. Considering the importance of the substrate of the active site in anchoring and electron transfer, Zhang et al. proposed a holey N‐doped graphene (HNG), and successfully anchored the Mo SAs on the HNG framework (Figure 4D) [47]. The HNG can effectively disperse and anchor the Mo atoms to form the Mo−N*_x_* moieties, which can highly boost the NRR activity and selectivity. It is worth mentioning that the Mo/HNG shows an excellent FE of 50.2% for the NH_4_^+^/NH_3_ production. As an electron‐rich center, oxygen vacancies also show great potential in the electrochemical energy conversion process owing to their potential in activating surface adsorbed molecules and altering surface electronic structures to boost reaction kinetics [48, 49]. Fang et al. anchored the intrinsic oxygen vacancy‐rich CeO_2_ nanoparticles on the MoN nanosheets (Figure 4E), and demonstrated that the electron‐deficient area of the nitrogen vacancy site can be enlarged by the synergistic effect of CeO_2_ nanoparticles and MoN nanosheets, which is beneficial for the N_2_ fixation [50]. With the promoted N_2_ adsorption/activation and *NNH/*NH_3_ generation, this CeO_2_−MoN shows an outstanding NH_3_ yield rate of 27.5 µg h^−1^ mg^−1^ at −0.3 V_RHE_ (Figure 4F).

**FIGURE 5:**
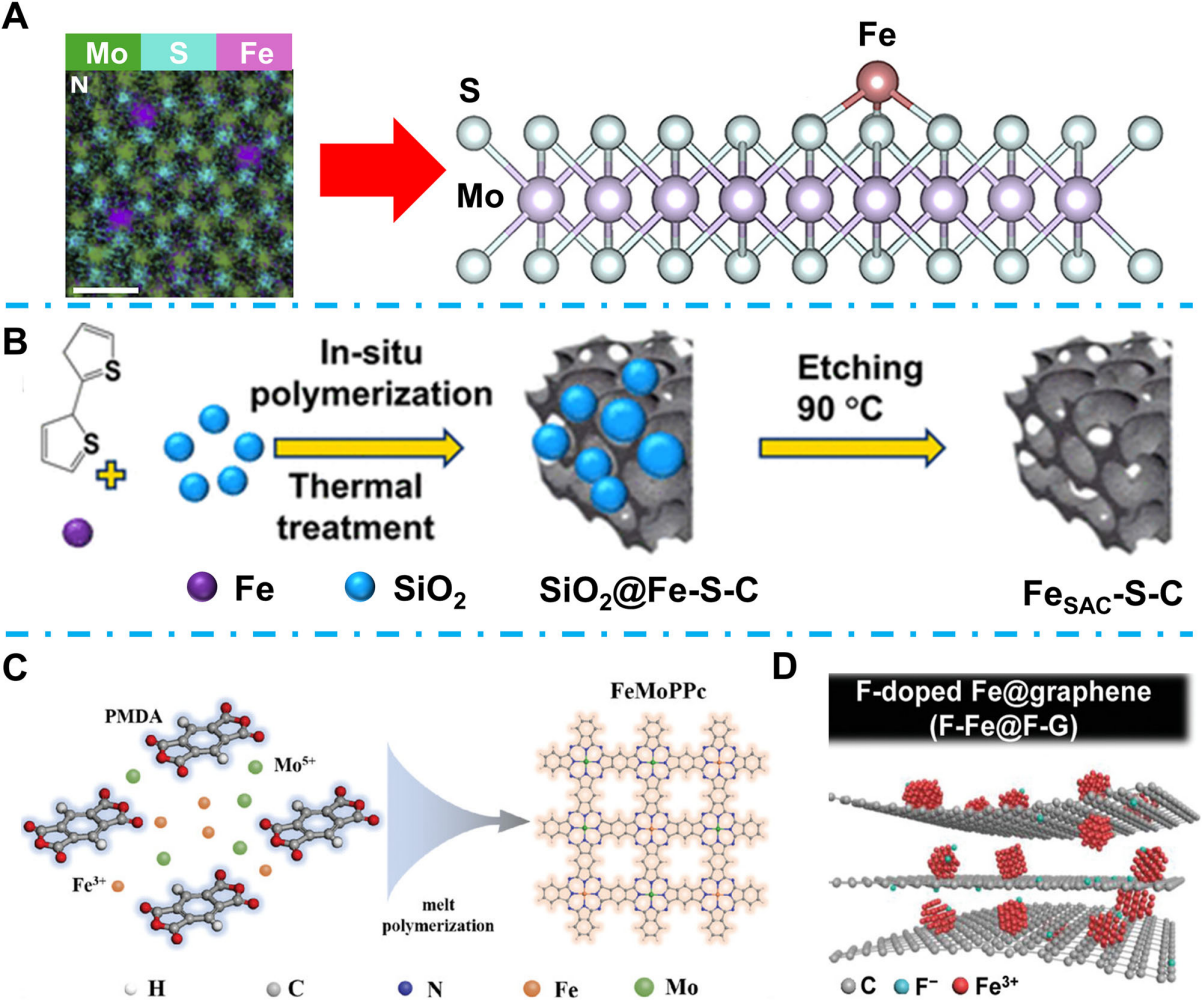
(A) EDX spectrum (left) and schematic illustration (right) of SACs‐MoS_2_‐Fe. Reproduced with permission [56]. Copyright 2020, Elsevier. (B) Fe_SAC_‐S‐C fabrication process schematic illustration. Reproduced with permission [58]. Copyright 2022, American Chemical Society. (C) Synthesis illustration of FeMoPPc [59]. Copyright 2021, Wiley‐VCH. (D) Structure diagram of F‐Fe@F‐G. Reproduced with permission [60]. Copyright 2022, Wiley‐VCH.

Fe is another non‐noble metal that emerged with excellent NRR catalytic activity owing to its top position in the volcano diagram for N_2_ fixation [51]. Many different Fe‐based catalysts have been reported for EC NRR to NH_3_, such as iron oxide [52], hydroxide iron oxide [53], iron carbide/nitride [54], and iron sulfide [55]. Li et al. point out that anchoring SACs on ultra‐thin 2D substrate can highly boost the NRR FE beneficial from the high curvature interfacial and high anchoring density on ultra‐broad 2D surface host [56]. In their research, they successfully fixed Fe SACs on the surface of an ultra‐thin MoS_2_ single layer (Figure 5A), while the anchored protruding Fe SACs can generate an electric field to polarize N_2_, which highly accelerated the electron transfer from catalysts to N_2_. As a result, their Fe‐MoS_2_ achieved a high NH_3_ yield rate of 36.1 mmol g^−1^ h^−1^ and FE of 31.6% at −0.2 V_RHE_. Zhu et al. suggested that structure‐stabilized FeOOH has excellent N_2_ fixation properties [53]. To overcome the intrinsic poor electronic conductivity of FeOOH, they decorated FeOOH QDs onto the graphene sheet, where the strong synergistic interaction can not only prevent the accumulation of FeOOH QDs but also boost the overall catalytic activity of EC NRR. The NH_3_ yield rate of their fabricated FeOOH QDs‐GS catalyst reached 27.3 µg h^−1 ^mg^−1^_cat_ with an FE of 14.6% at −0.4 V_RHE_. The decoration of active metal sites on heteroatom‐doped carbon substrate can lead to the regulated electronic structures of the active center for EC NRR, owing to optimized charge distribution resulting from the formed M−N linkages. However, high free energy is still required on the M–N–C surface for the NH*_x_* intermediates formation process [57]. Considering this, Ahmed et al. proposed a Fe–S–C linkage (Figure 5B), while the S can serve as a bridge for the C and Fe to generate localized sites, which can also serve as the centers for the N_2_ fixation and activation [58]. Further, the S can promote H adsorption, which is favorable for free energy and decreases the NRR process. Tuning the electronic state of catalytic sites can be an efficient strategy for NRR property optimization. Wang et al. designed a Fe–Mo hetero‐single‐atom catalyst FeMoPPc (Figure 5C), which contains separated FeN_4_ and MoN_4_ catalytic sites [59]. They found that the participation of Mo SAs led to the charge accumulation of Fe, which can not only promote the N_2_ fixation but also induce the Fe spin state from high to medium spin, promoting the first protonation step of NRR. Finally, this medium‐spin Fe active site achieved two times enhancement on NRR FE compared to the high‐spin catalyst. Fluorine, which possesses strong electronegativity, is considered an ideal candidate for modulating the surface electronic state of Fe‐based catalysts. However, because of the strong electronegativity, fluorine can easily react with Fe to form iron fluoride, which is harmful for the N_2_ fixation of NRR. Wang et al. fabricated an F‐doped Fe@graphene catalyst (Figure 5D) through a potassium vapor treatment. The F atoms can significantly induce the surface charge redistribution of the Fe active sites, leading to the optimized N_2_ absorption. As a result, their F‐doped Fe@graphene showed efficient NRR property with high FE of 41.6% and 53.3 µg mg^−1^ h^−1^ NH_3_ yield rate [60].

Apart from Mo and Fe, some other transition metal‐based catalysts have been investigated for EC NRR. Ti‐based catalysts are regarded as excellent NRR electrocatalysts beneficial from their preferential affinity for N [61]. Zhao et al. proposed a Li‐TiO_2_ electrocatalyst for EC NRR. The Li‐intercalation strategy was applied to TiO_2_ nanosheets, while the structural distortion can enhance the oxygen vacancy of Li‐TiO_2_. The activated Li‐TiO_2_(B) nanosheets eventually showed a more than six‐fold improvement in EC NRR property, demonstrating a high FE of 18.2% at −0.4 V_RHE_ [61]. V‐based catalysts have also been investigated for EC NRR inspired by the V‐dependent nitrogenases. Wu et al. proposed a V‐doped TiO_2_ nanorod (V‐TiO_2_) while V served as an excellent dopant for TiO_2_‐based N_2_‐to‐NH_3_ fixation, which achieved a high FE of 15.3% at −0.4 V_RHE_ [62]. Mn can be another dopant for TiO_2_ owing to its multiple oxidation states, which could modulate the concentration of oxygen vacancy. Chen et al. proposed Mn‐TiO_2_ electrocatalyst and demonstrated that the enhanced oxygen vacancy and the corresponding generated bi‐Ti^3+^ pair can promote the N_2_ activation and lead to boosted NRR property [63]. Wen et al. proposed a mesoporous MnO_2_ nanosheet for EC NRR and demonstrated that the Mn^3+^–Mn^3+^ pairs with high spin state can effectively promote the NRR activity, which resulted in a boosted NH_3_ yield rate of 147.2 µg h^−1^ mg_cat_^−1^ at −0.75 V_RHE_ [64]. Kang et al. proposed that the p‐orbital metals can be good candidates for EC NRR with suitable modulation. They modulated the Bi 6p energy levels by downsizing the Bi clusters to the atomic level, which highly boosted the catalytic sites for N_2_ adsorption and activation. Their BiO*_x_* eventually achieved a high NH_3_ yield rate of 113 µg h^−1^ mg^−1^_cat_ with outstanding FE of 30% [65]. Goyal et al. proposed that an excellent NH_3_ electrosynthesis mediator is required to meet several conditions, which include spontaneous formation of a nitride, facile N_2_ activation, stable N vacancy, exergonic binding of N vacancy and N_2_, and salts of the mediator solubility in nonaqueous electrolyte. Their research demonstrated that Ca‐mediated NH_3_ electrosynthesis can also be a good strategy. The electrodeposited Ca can react with N_2_ spontaneously to form Ca_3_N_2,_ which can serve as a mediator for EC NRR. Finally, Ca showed a high FE of 50% at −15 mA cm^2^ [66].

#### 2.1.2 TMCs for EC NORR

**FIGURE 6:**
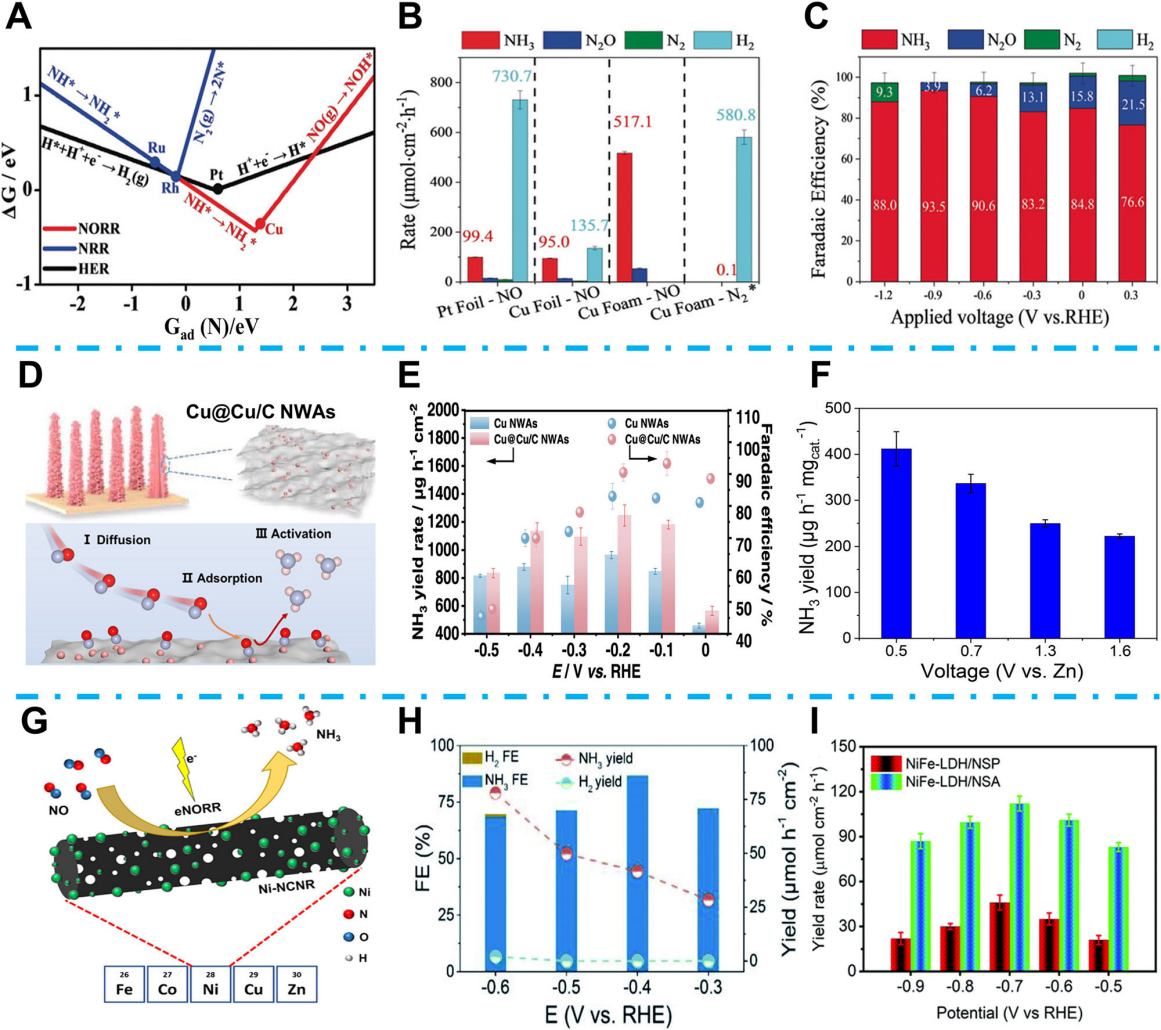
(A) Δ*G*‐determining steps comparison for different ammonia synthesis pathways. Reproduced with permission [67]. Copyright 2020, Wiley‐VCH. (B) Different products yield different rates of Cu foam for NORR. Reproduced with permission [67]. Copyright 2020, Wiley‐VCH. (C) NORR FE of Cu foam with different applied bias. Reproduced with permission [67]. Copyright 2020, Wiley‐VCH. (D) Schematic illustration of Cu@Cu/C NWAs catalyst. Reproduced with permission [69]. Copyright 2024, American Chemical Society. (E) EC NORR FE and NH_3_ yield rate at different potentials for Cu@Cu/C NWAs catalyst. Reproduced with permission [69]. Copyright 2024, American Chemical Society. (F) NH_3_ yield rate of MoS_2_/CP cathode at different potentials. Reproduced with permission [70]. Copyright 2021, Wiley‐VCH. (G) Schematic illustration of Ni‐NCNR catalyst. Reproduced with permission [73]. Copyright 2023, American Chemical Society. (H) NH_3_ yield rate and FE at different potentials for Fe_2_O_3_/CP catalyst. Reproduced with permission [74]. Copyright 2022, Royal Society of Chemistry. (I) NH_3_ yield rate of NiFe‐LDH/NSA at different potentials. Reproduced with permission [75]. Copyright 2022, Royal Society of Chemistry.

Cu‐based materials were considered the most promising EC NORR to NH_3_ synthesis catalyst. Using theoretical calculation, Long et al. recently demonstrated that among different transition metals, their NRR catalytic activity is consistently lower than HER, resulting in poor NH_3_ product selectivity (Figure 6A) [67]. Nevertheless, NORR shows a more favorable energy diagram than HER, while Cu (111) shows the best catalytic property of NORR to NH_3_ synthesis. To support the computational calculation, they compared the NORR property of Pt and Cu foil, as shown in Figure 6B. Although Pt foil showed a similar NH_3_ yield rate compared to that of Cu foil, Pt exhibited much higher selectivity of H_2_ generation, which demonstrated a poor FE of NORR to NH_3_. Furthermore, their Cu foam catalyst achieved a high NH_3_ yield rate of 517.1 µmol cm^−2^·h^−1^ at −0.9 V_RHE_, and achieved high FE over 80% at voltages below 0 V_RHE_ (Figure 6C). Ren et al. proposed that topological quantum Cu_2_Si can be applied for efficient NORR to NH_3_, while they found that NORR to NH_3_ formation was spontaneous without any energy barrier, which result from the enhanced NO adsorption and optimized hydrogenation [68]. Meng et al. proposed that for the gas‐involved NORR, the poor NO solubility is another nonnegligible issue for the efficient mass transfer among the gas–solid–liquid interface [69]. Although concentrating on the NO gas can improve this problem, the accompanying economic issue can not be ignored. To achieve efficient NORR with low NO concentration, Meng et al. proposed a porous carbon‐supported Cu@Cu/C catalyst (Figure 6D); the porous carbon supports with abundant channels can enhance the local NO concentration while the metal–support interaction between separated Cu clusters and porous carbon supports can accelerate the *NO and *H adsorption. Finally, their Cu@Cu/C NWAs attained high NH_3_ yield rate of 1180.5 µg h^−1^ cm^−2^ and FE of 93% at 3% NO concentration (Figure 6E) [69]. Zhang et al. applied MoS_2_ nanosheet onto graphite felt (MoS_2_/GF) for EC NORR to NH_3_ synthesis [70]. They found that the charged Mo‐edge can accelerate the NO adsorption/activation and inhibit the N─N coupling. In acidic conditions, their Zn‐NO battery, which MoS_2_/GF serves as a cathode, achieved a high NH_3_ yield of 411.8 µg h^−1^ mg_cat_^−1^ (Figure 6F). Meng et al. designed N‐doped C nanosheets supported MoC catalyst (MoC/NCS), while the strong electronic interaction between MoC (1 1 1) and NO can highly reduce the energy barrier of NORR [71]. Strikingly, their MoC/NCS demonstrated a high NH_3_ yield rate of 1350 µg h^−1^ cm^−2^ at −0.8 V_RHE_ with a high FE of 89%. Muthusamy et al. also chose C fiber to support and fabricated a nitrogen‐carbon‐wrapped metallic Ni (NiNC@CF) catalyst for EC NORR. The intense interaction between CF and zero‐valent Ni resulted in a high FE of 87% under saturated NO electrolyte [72]. Dhanabal et al. decorated transition metals onto 1D N‐doped carbon nanorods (NCNR) and investigated their EC NORR property (Figure 6G) [73]. Their research demonstrated that Ni showed the best property among different transition metals. In addition, they also found that different annealing temperatures can lead to regulated graphitization and porosity of the Ni‐NCNR, which resulted in optimized NORR properties. Iron‐based materials also have been reported as catalysts for NORR to NH_3_ production. Liang et al. fabricated Fe_2_O_3_ nanorods on carbon paper, and they discovered that an “acceptance‐donation” mechanism led to strong NO adsorption on Fe_2_O_3_ (1 0 4) surface, together with the 2π* back‐donation effect, resulting in a high NH_3_ yield rate of 78.02 µmol h^−1^ cm^−2^ with a FE of 86.7% (Figure 6H) [74]. Double metal‐layered double hydroxide (LDH) can also be applied for EC NORR owing to the mutual adjustment between the metal ions and the lamellar structure. Meng et al. proposed a NiFe‐LDH nanosheet array (NiFe‐LDH/NSA) on Ni foam and achieved highly efficient EC NORR to NH_3_ under ambient conditions [75]. Applying Ni foam can relieve the utilization of binder and boost the charge transfer during the NORR. As a result, their NiFe‐LDH/NSA delivered a high NH_3_ yield rate of 112 µmol h^−1^ cm^−2^ (Figure 6I).

#### 2.1.3 TMCs for EC NtrRR

**FIGURE 7:**
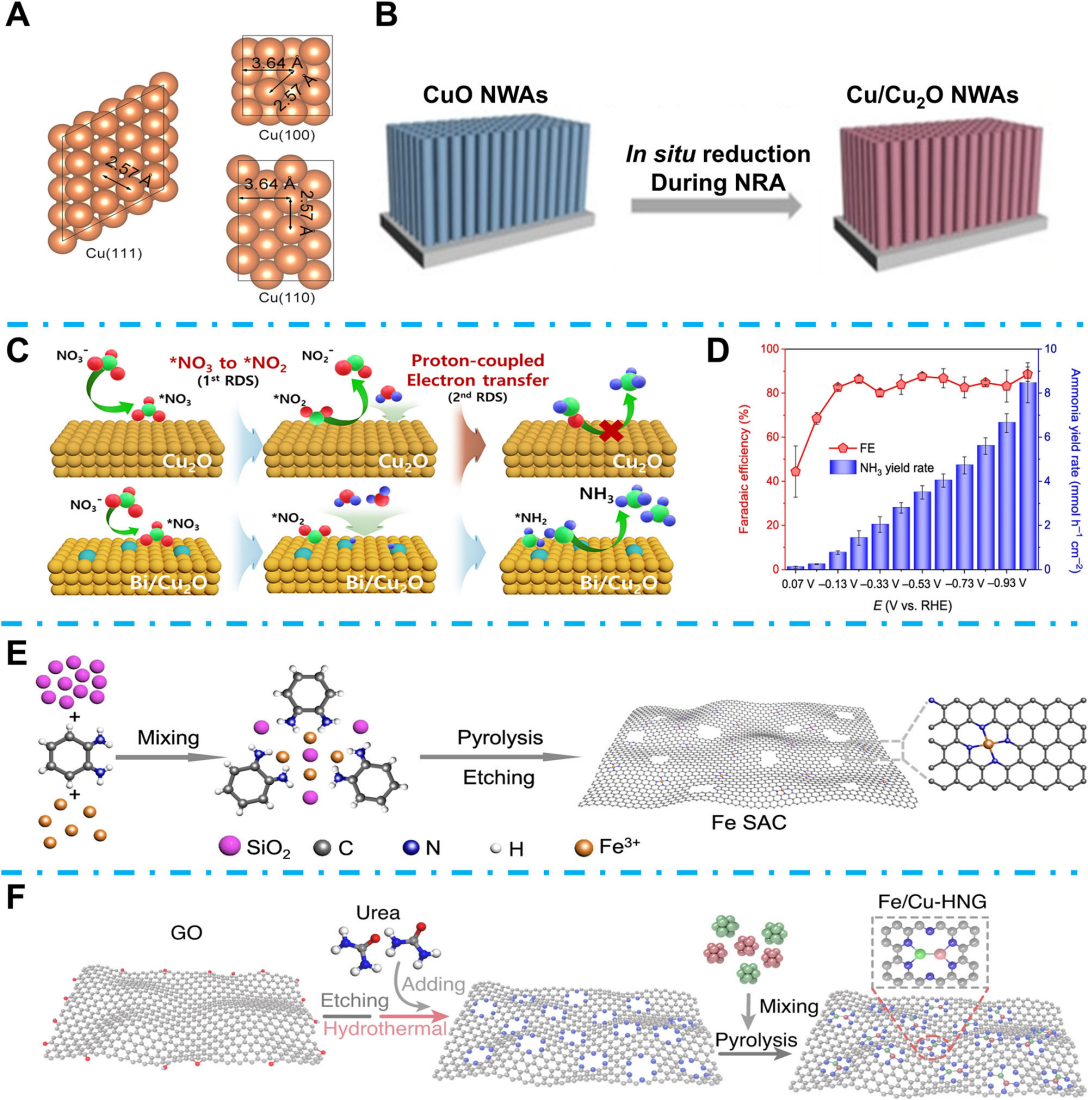
(A) Surface structure of Cu with different crystalline faces. Reproduced with permission [77]. Copyright 2021, American Chemical Society. (B) Schematic illustration of CuO in situ reconstruction during EC NtrRR. Reproduced with permission [79]. Copyright 2020, Wiley‐VCH. (C) Schematic illustration of the Bi/Cu_2_O EC NtrRR mechanism. Reproduced with permission [80]. Copyright 2024, Elsevier. (D) NtrRR to NH_3_ yield rate and FE at different potentials for CoP‐CNS. Reproduced with permission [81]. Copyright 2022, Springer Nature. (E) Schematic illustration of the Fe SAC fabrication process. Reproduced with permission [82]. Copyright 2021, Springer Nature. (F) Schematic illustration of Fe/Cu‐HNG construction. Reproduced with permission [83]. Copyright 2023, Springer Nature.

Among different non‐noble metals, Cu shows the most active surface for NtrRR and has been widely researched as an NtrRR catalyst [76]. Hu et al. compared the NtrRR to NH_3_ property on different copper metal crystal surfaces, as shown in Figure 7A, including Cu (1 1 1), (1 0 0), and (1 1 0) [77]. Their research demonstrated that the competition of NtrRR and HER was highly dependent on pH value and crystal surfaces, while the Cu (1 0 0) possessed the widest pH range of ≈1.42–14 for NtrRR, Cu (1 1 1) was suitable in neutral or basic conditions. For the polycrystalline Cu catalysts, the (1 0 0) surface serves as the active site in acid conditions, while (1 1 1) surface works in pH conditions over 5.63. Inspired by the research on nitrate reductase, Chen et al. discovered that the combination of high‐activity metal active sites and protease scaffolds could effectively boost the NtrRR to NH_3_ property [78]. The 3,4,9,10‐perylenetetracarboxylic dianhydride (PTCDA) organic molecule was chosen as the scaffold, and a series of metals were doped with PTCDA. They found that Cu shows the highest total FE of NtrRR and excellent selectivity for NH_3_ formation. Interestingly, the metals Co, Fe, and Ni also showed excellent FE; however, Cu still had significant advantages in catalytic activity for the EC NtrRR [78]. CuO nanowire arrays (NWAs) were also considered excellent NtrRR catalysts. Wang et al. applied CuO NWAs for EC NtrRR, and they found that during EC NtrRR, CuO can be in situ reduced to Cu/Cu_2_O, which serves as active sites (Figure 7B) [79]. The electron transfer between the Cu and Cu_2_O interface can efficiently suppress the HER and promote the *NOH NtrRR intermediate formation. As a result, their CuO NWA achieved a high NH_3_ yield rate of 0.2449 mmol h^−1^ cm^−2^ with an FE of 95.8%. P‐orbital metal doping into a d‐orbital Cu‐based catalyst could enhance the catalytic activity and EC NtrRR to NH_3_ selectivity. Our group introduced p‐orbital Bi on the surface of Cu_2_O, while Bi can not only inhibit HER, but also optimize the *NO_2_ intermediate adsorption (Figure 7C), which can promote the second rate‐determining step of EC NtrRR, resulting in boosted NtrRR to NH_3_ selectivity. Consequently, the Bi/Cu_2_O achieved a significant FE of 99.2% with a high NH_3_ yield rate of 2562.56 µg h^−1^ ${\mathrm{mg}}_{\mathrm{cat}}^{-1}$ [80]. Transition metal phosphates can effectively accelerate the kinetics of protonation reactions. Fan et al. fabricated the CoP nanosphere on a carbon nanosheet array (CoP‐CNS) used for EC NtrRR [81]. Their research demonstrated that to achieve a high NtrRR to NH_3_ yield rate and high FE, it is not advisable to inhibit HER alone traditionally, but a dynamic balance of active H ions production and consumption should be maintained in the NtrRR system. Finally, their CoP‐CNS achieved a skyscraping NH_3_ yield rate of 8.47 mmol h^−1^ cm^−2^ and maintained a high FE of over 80% for 123 h (Figure 7D) [81]. Wu et al. proposed that the SACs can suppress the NtrRR to N_2_ pathway and promote the selectivity of NH_3_ formation [82]. They deposited Fe SAC on a standard glassy carbon electrode (Figure 7E), demonstrating that high‐efficient Fe‐N_4_ active centers can decrease the thermodynamic barrier of NtrRR. Finally, their Fe SACs led to a high NH_3_ yield rate of 0.46 mmol h^−1^ cm^−2^ with a FE of 75%. Zhang et al. proposed that for the multi‐electron transfer NtrRR, one specific SAC active site still faced challenges in the adsorption of multiple intermediates, while applying dual active sites can effectively expand the coordination environment of catalysts [83]. In their research, N‐doped graphene (HNG) support was first etched as a rich structure. They successfully anchored Fe and Cu dual‐metal atoms on the holey edge and achieved a dimer structure in the HNG holes (Figure 7F). The strong interaction between d‐orbitals of Fe/Cu site and NO_3_^−^ could accelerate the first anionic adsorption process, while the Fe/Cu heterostructure led to high selectivity of NtrRR to NH_3_. The synergistic effects of Fe/Cu dual active sites finally demonstrated an excellent NH_3_ yield rate of 1.08 mmol h^−1^ mg^−1^ at −0.5 V_RHE_ and a high FE of 92.51% at −0.3 V_RHE_ [83]. Lee et al. designed a zinc‐nitrate (Zn‐NO_3_^−^) battery system that can achieve HN_3_ formation and electricity generation simultaneously. An ultrafast CO_2_ laser irradiation was applied to the CoFe Prussian blue analogs to fabricate hollow CoFe_2_O_4_@NC nanocubes. And they demonstrated that the formed high‐valent Fe/CoOOH on the surface of CoFe_2_O_4_@NC during NtrRR optimized the key intermediates (*NO_2_/*NO) adsorption energy, which eventually achieved a high NH_3_ yield rate of 10.9 mg h^−1^ cm^−2^ at −0.47 V_RHE_. Moreover, this Zn‐NO_3_^−^ battery showed dual functionality, achieving electricity generation with NH_3_ production with a stable open‐circuit voltage [84]. Zhou et al. designed another Zn‐NO_3_^−^ battery system by using a 2D Cu plate‐based electrocatalyst. They proposed that the nanosized 2D Cu sheet can facilitate a steady fluid field, which is beneficial for catalytic interface refreshing, providing high‐rate EC NtrRR in the Zn‐NO_3_^−^ battery. Combining with surface gas engineering, their Zn‐NO_3_^−^ battery achieved a high NH_3_ FE of 85.4% with a high power density of 12.09 mW cm^−2^ [85].

### 2.2 TMCs for PEC NH_3_ Synthesis

#### 2.2.1 TMCs for PEC NRR

**FIGURE 8:**
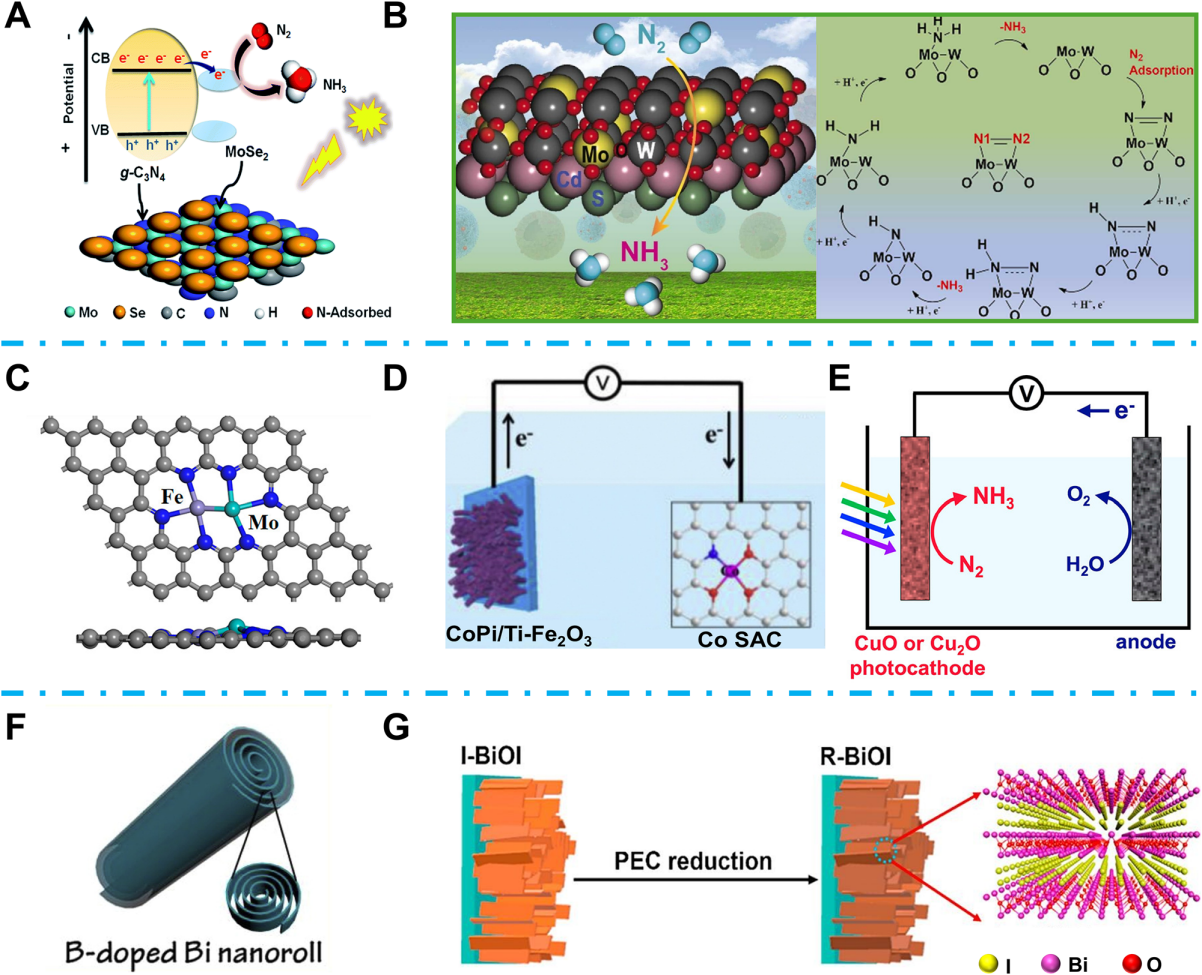
(A) Schematic illustration of MoSe_2_@g‐C_3_N_4_ heterojunctions for PEC NRR. Reproduced with permission [89]. Copyright 2021, Royal Society of Chemistry. (B) Schematic illustration of Mo‐doped WO_3_@CdS (left) and mechanism (right) for PEC NRR on the surface of Mo‐doped WO_3_. Reproduced with permission [91]. Copyright 2022, Elsevier. (C) Schematic illustration of the FeMo/graphene structure. Reproduced with permission [92]. Copyright 2021, American Chemical Society. (D) Schematic illustration of CoPi/Ti‐Fe_2_O_3_@Co SAC PEC NRR system. Reproduced with permission [93]. Copyright 2021, Elsevier. (E) Schematic illustration of CuO or Cu_2_O PEC NRR system. Reproduced with permission [94]. Copyright 2020, American Chemical Society. (F) Schematic illustration of B‐doped Bi nanoroll. Reproduced with permission [95]. Copyright 2020, Elsevier. (G) Schematic illustration of R‐BiOI synthesis process. Reproduced with permission [96]. Copyright 2019, Elsevier.

TiO_2_ has been widely researched as a PEC energy conversion catalyst owing to its excellent optical properties and environmental friendliness. However, the fast photo‐induced charge recombination seriously restricted its PEC energy conversion efficiency [86]. Ye et al. proposed that incorporating a suitable cocatalyst can efficiently boost the charge separation and enhance the solar‐to‐chemical selectivity and catalytic activity [87]. Inspired by nitrogenases, they assembled 2D MoS_2_ with TiO_2_ and applied the MoS_2_@TiO_2_ for PEC NRR, which achieved a high NH_3_ yield rate of 1.47 × 10^−6^ mol h^−1^ cm^−2^ at −0.3 V_RHE_. Li et al. introduced MoC into the PEC NRR system. To overcome the poor conductivity of MoC, they covered graphitized carbon on MoC and prepared core‐shell Mo_2_C/C nanoparticles, which achieved a promoted NH_3_ yield rate of 6.6 µg h^−1^ mg^−1^ [88]. Layered MoSe_2_ with a suitable bandgap can also be applied for PEC NRR. Mushtaq et al. coupled MoSe_2_ with 2D graphite‐like g‐C_3_N_4_ and successfully fabricated hierarchical MoSe_2_@g‐C_3_N_4_ hybrids (Figure 8A) [89]. The synergistic interactions between MoSe_2_ and g‐C_3_N_4_ can enrich the active sites, while the hierarchical structure provided enhanced light absorption, which achieved an optimal NH_3_ yield rate of 7.72 µmol h^−1^ cm^−2^. The tungsten element is also demonstrated to activate N≡N [90]. Mushtaq et al. decorated Mo on WO_3_ (Mo‐WO_3_), then CdS nanoparticles were in situ decorated on the Mo‐WO_3_ to fabricate hierarchical Mo‐WO_3_@CdS‐*x* polyporous hollow microspheres (Figure 8B) [91]. The polyporous hollow structure can enlarge the surface area and boost light absorption, while the ultra‐thin shell can reduce the photo‐induced charge migration length, accelerating the PEC NRR activity. Furthermore, the W–Mo dimer unfolded stronger N_2_ adsorption than the W−W dimer, which served as N_2_ adsorption sites (Figure 8B), and the heterogeneity of W−Mo led to the polarized N_2_ molecules finally achieving a fast NH_3_ yield rate of 38.99 µg h^−1^ mg_cat_^−1^ at −0.3 V_RHE_ [91]. Wang et al. also investigated the properties of heteronuclear metal dimers in PEC NRR. Twenty‐eight bimetal combinations were embedded in g‐C_3_N_4_, and they found that FeMo/g‐C_3_N_4_ (Figure 8C) appeared to have a favorable negative limiting potential [92]. Moreover, they found that the metal dimers Ti/Mo, W/Mo, and Ni/Mo can be excellent candidates for PEC NRR owing to their suitable band edge positions. Wang et al. developed a PEC NRR system consisting of CoPi/Ti‐Fe_2_O_3_ photoanode and Co‐SAC cathode (Figure 8D) [93]. During the PEC NRR process, OER occurred at the anode part, while the photoinduced electrons were transferred to the cathode part for NH_3_ generation. As a result, the NH_3_ yield rate of their PEC NRR system reached 1021.5 µg mg_Co_^−1^ h^−1^ at 1.2 V_RHE_. Jang et al. investigated the application of CuO and Cu_2_O as photocathodes in PEC NRR (Figure 8E) [94]. The suitable band gap energies of CuO (≈1.5 eV) and Cu_2_O (≈2.0 eV) lead to efficient light absorption in the visible range, and their insensitive activity in HER makes them feasible in PEC NRR. Finally, both CuO and Cu_2_O showed excellent PEC NRR to NH_3_ FE (CuO 17% and Cu_2_O 20%) [94]. Xu et al. proposed a B‐doped Bi nanoroll (BDB NR) catalyst for the PEC NRR system (Figure 8F) [95]. TiO_2_ was a photoanode to provide photogenerated electrons, while the BDB NR served as a cathode for NH_3_ production. The B doping highly reduced the energy barrier of N_2_ → *NNH NRR step while the nanoroll facilitated the N_2_ adsorption, which led to an efficient NH_3_ yield rate of 29.2 mg_NH3_ g_cat_^−1^ h^−1^ at 0.48 V_RHE_ for their PEC NRR system [95]. Bai et al. proposed that surface defect engineering can enrich Lewis‐base surface chemical environment, which can accelerate the N_2_ adsorption and suppress HER. They reported an interlayered reduced BiOI (R‐BiOI) with enriched surface oxygen vacancies for PEC NRR (Figure 8G) [96]. The oxygen vacancies can serve as carrier trap states and suppress the charge recombination. Finally, their R‐BiOI photocathode achieved a desirable NH_3_ yield rate of 1.4 mmol m^−2^ h^−1^ [96].

#### 2.2.2 TMCs for PEC NORR

**FIGURE 9:**
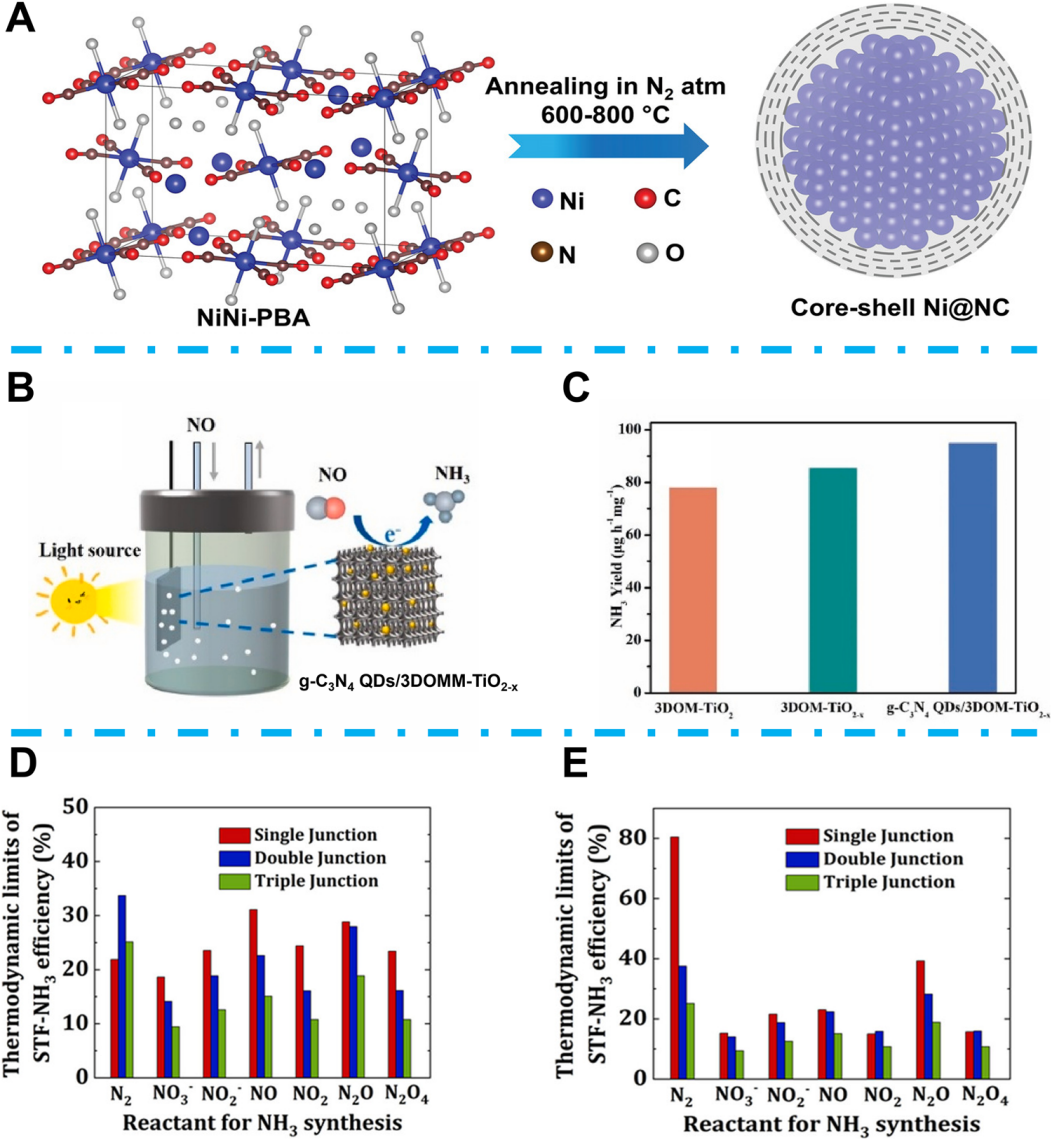
(A) Schematic illustration of the Ni@NC synthesis process. Reproduced with permission [97]. Copyright 2022, Wiley‐VCH. (B) Schematic illustration of g‐C_3_N_4_ QDs/3DOMM‐TiO_2−_ *_x_* PEC NORR system. Reproduced with permission [98]. (C) The photocatalytic NH_3_ yield rates. Reproduced with permission [98]. Copyright 2023, Elsevier. (D) Solar to fuel efficiency of PEC NH_3_ synthesis by using different *N* sources while the water oxidation reaction was conducted at the counterpart. Reproduced with permission [99]. (E) Solar to fuel efficiency of PEC NH_3_ synthesis by using different *N* sources while the hydrogen oxidation reaction was conducted at the counterpart. Reproduced with permission [99]. Copyright 2022, American Chemical Society.

The development of EC NORR has received much attention. However, the research of PEC NORR for NH_3_ formation is still in its infancy. Markandaraj et al. indicate that an ultra‐thin carbon‐clad metal core‐shell structure can provide excellent electrocatalytic properties and stability for the PEC NORR process [97]. They proposed an N‐doped porous C‐coated Ni (Ni@NC) catalyst and applied it for PEC NORR (Figure 9A). The ultrathin porous NC shell can prevent Ni from dissolution, accelerate charge and NO transport during the NORR process. The as‐prepared Ni@NC cathode was assembled with RuO_2_ anode for PEC NORR. During the 3 h measurement, the photocurrent density remained constant at around 6 mA cm^−2^ with a bias of 2.1 V under 1 sun illumination, while the PEC NORR to NH_3_ FE attained 50% and solar to NH_3_ efficiency reached 1.7% [97]. Chen et al. proposed a 3D porous g‐C_3_N_4_ QDs/3DOMM‐TiO_2−_ *_x_* catalyst with oxygen vacancies and Ti^3+^ [98]. As shown in Figure 9B, the fabricated g‐C_3_N_4_ QDs/3DOMM‐TiO_2−_ *_x_* was applied as a photocathode for PEC NORR. The researchers indicated that introducing oxygen vacancies and Ti^3+^ can reduce the band gap and enhance the selectivity of PEC NORR to NH_3_. As a result, this g‐C_3_N_4_ QDs/3DOMM‐TiO_2−_ *_x_* photocathode enhanced NH_3_ yield rate of 95.07 µg h^−1^ mg^−1^ (Figure 9C) [98]. Kani et al. investigated the design of a light absorber in the PEC NH_3_ synthesis system with different nitrogen sources by theoretical calculation [99]. In particular, three types of light absorbers, including single, double, and triple junctions were analyzed to find a satisfactory solar to NH_3_ efficiency for PEC NORR from different *N* sources. They found that for PEC NORR, the single junction light absorber showed the best theoretical solar to NH_3_ efficiency over 30% while coupled with OER at the anode part. Meanwhile, for that of PEC NRR, the double junction light absorber showed the best property (Figure 9D). Differently, while the PEC NH_3_ production reaction was coupled with H_2_ oxidation, the single junction light absorber showed the best efficiency for both PEC NRR and PEC NORR, but PEC NRR demonstrated significant advantages over PEC NORR (Figure 9E) [99].

#### 2.2.3 TMCs for PEC NtrRR

**FIGURE 10:**
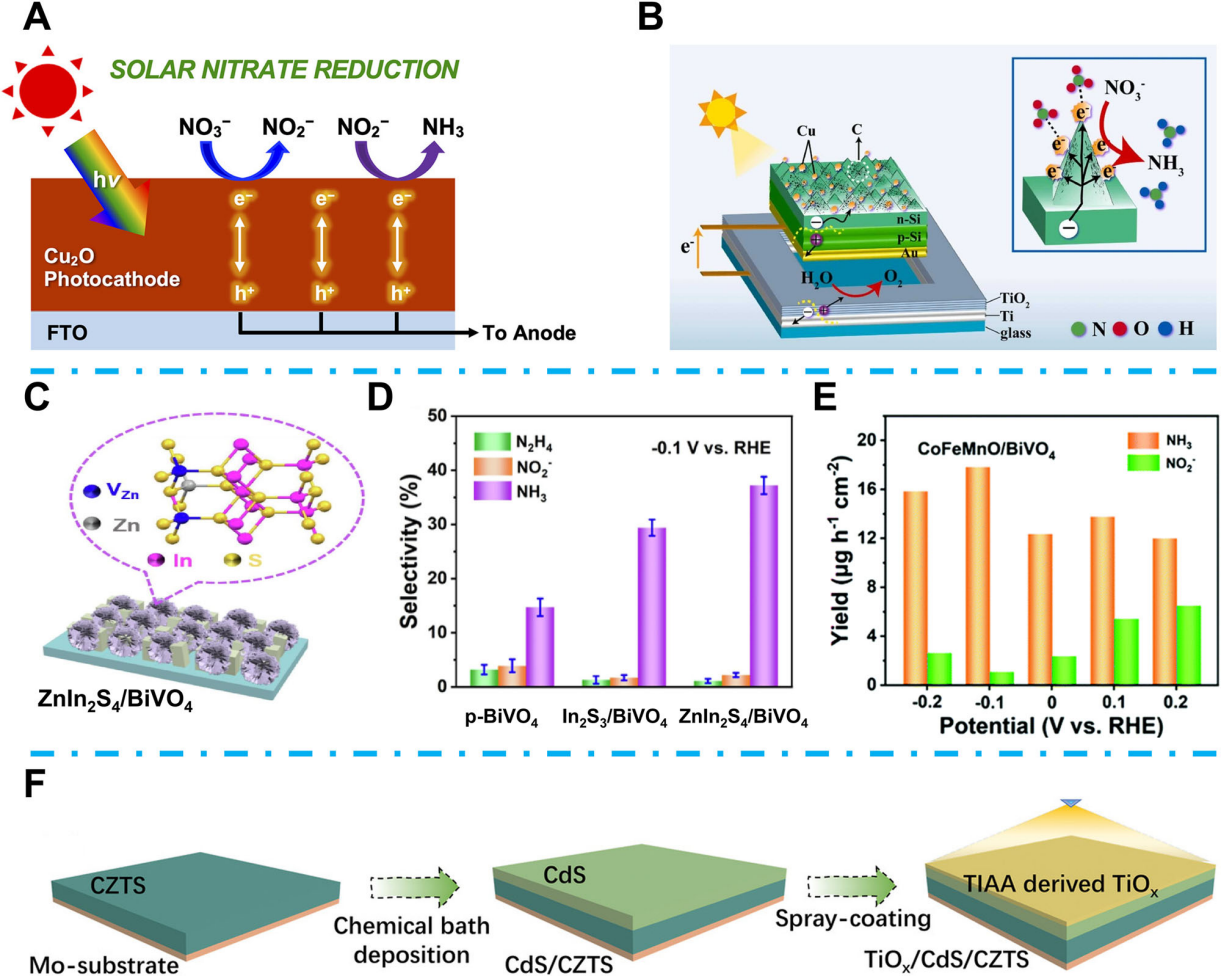
(A) Schematic illustration of Cu_2_O photocathode for PEC NtrRR. Reproduced with permission [100]. Copyright 2024, American Chemical Society. (B) Schematic illustration of Cu/C/Si‐TiO_2_. Reproduced with permission [102]. Copyright 2024, Elsevier. (C) The structure of ZnIn_2_S_4_/BiVO_4_. Reproduced with permission [103]. (D) PEC NtrRR products selectivity of ZnIn_2_S_4_/BiVO_4_. Reproduced with permission [103]. Copyright 2022, Elsevier. (E) NH_3_ yield rate based on CoFeMnO/BiVO_4_ at different potentials. Reproduced with permission [104]. Copyright 2022, Royal Society of Chemistry. (F) Schematic illustration of the TiO*_x_*/CdS/CZTS photocathode synthesis process. Reproduced with permission [107]. Copyright 2022, Wiley‐VCH.

For the NtrRR to NH_3_ production, the first conversion step from nitrate to nitrite requires a high overpotential of 0.8 V, considered the rate‐limiting step of EC NtrRR. Therefore, if the conversion from nitrate to nitrite can be realized spontaneously using solar energy, the NtrRR to NH_3_ conversion catalytic property would be highly promoted [100, 101]. Kim et al. reported a Cu_2_O photocathode and applied for PEC NtrRR (Figure 10A) [100]. They indicated that the suitable conduction band minimum position of Cu_2_O (−0.8 V_RHE_) led to high overpotential (>1.6 V) of photo‐induced electrons in the conduction band, which is sufficient for the conversion from nitrate to nitrite, without extra energy requirement. As a result, their Cu_2_O photocathode confirmed a high nitrate to nitrite FE of 85% and 50% nitrite to NH_3_ FE [100]. Ding et al. proposed a Cu nanoparticle decorated hierarchically structured Si‐based photocathode and assembled a PEC NtrRR device with a bulk TiO_2_ photoanode (Figure 10B) [102]. They indicated that the photo‐generated electrons during the PEC NtrRR process can lead to enhanced Lewis acid sites on Cu, accelerating nitrate adsorption and hydrogenation. Finally, the PEC NtrRR NH_3_ yield rate Cu/C/Si photocathode reached 115.3 µmol h^−1^ cm^−2^ [102]. P‐type BiVO_4_ is also considered a candidate as a photocathode for PEC NtrRR owing to its suitable bandgap. However, the side reactions and competitive HER seriously restricted its NH_3_ selectivity and yield activity. Wang et al. indicated that the introduction of frustrated Lewis pairs (FLPs, Lewis acid, and Lewis base) can activate the electron acceptor and donation process during the PEC NtrRR [103]. In their research, they coupled the P‐type BiVO_4_ with hexagonal layered ZnIn_2_S_4_, which possessed affluent zinc vacancies (Figure 10C). The broken continuity of Zn–S resulted from the zinc vacancies, which achieved the introduction of FLPs, resulted in boosted nitrate adsorption. Furthermore, the exposed Lewis base can enhance the charge transfer and boost the NtrRR catalytic activity. The ZnIn_2_S_4_/BiVO_4_ finally obtained a promoted PEC NtrRR to NH_3_ yield rate of 29.95 µg h^−1^ cm^−2^ and considerable NH_3_ selectivity of 37.6% (Figure 10D) [103]. Fan's group applied another high entropy CoFeMnO cocatalyst on P‐type BiVO_4_ while introducing high entropy amorphous metal oxide, which can enhance the charge density and nitrate adsorption [104]. As a result, the CoFeMnO/BiVO_4_ demonstrated excellent PEC NtrRR to NH_3_ selectivity and confirmed a best NH_3_ yield rate of 17.82 µg h^−1^ cm^−2^ at −0.1 V_RHE_ (Figure 10E). In recent years, Cu_2_ZnSnS_4_ (CZTS)‐based photocathodes have been widely used in PEC energy conversion owing to its satisfactory light absorption property, environmental and economical friendliness [105]. In addition, the introduced p–n junction of CZTS with CdS can efficiently inhibit charge recombination during the PEC catalytic process [106]. Zhou et al. reported a rationally designed TiO*_x_*/CdS/CZTS photocathode with optimized surface defect sites for PEC NtrRR (Figure 10F) [107]. They demonstrated that the ratio of surface defect sites on TiO*_x_* layer was closely related to the nitrate and *NO_2_ adsorption, and the optimized TiO*_x_*/CdS/CZTS confirmed a high nitrate to NH_3_ FE of 89.1% at 0.38 V_RHE_. Xu et al. investigated the influence of oxygen defect on TiO_2_ for PEC NtrRR [108]. Oxygen defect enriched ultra thin TiO_2_ layer was coated on p‐type SiO_2_, while the oxygen defect can efficiently enhance the nitrate adsorption and reduce the PEC NtrRR energy barrier, which led to a high NH_3_ yield FE over 94% [108]. Kani et al. reported an oxide‐derived Co (OD‐Co) NtrRR catalyst, and assembled the EC cell with a GaInP/GaAs/Ge solar cell for PEC ammonia synthesis. Finally, this PV‐electrolyzer cell achieved a significant solar to ammonia efficiency of 11% [109].

Overall, transition metal‐based materials have been widely explored as catalysts for EC and PEC ammonia synthesis, and remarkable achievements have been reported, which confirmed the feasibility of economical and environmentally friendly artificial ammonia synthesis. We summarized significant achievements of EC (Table 1) and PEC (Table 2) ammonia synthesis based on TMCs in recent years. Specifically, to achieve efficient and economical practical ammonia synthesis in the future, the catalyst design under aqueous conditions is required to meet the strong competition against HER, and possess excellent reactant adsorption capacity. Meanwhile, for NRR, the high N≡N bond energy is the key point to design highly active catalysts; for NORR, low NO concentration in exhaust gas should be considered for the catalyst exploration; while for NtrRR, the practical application of TMCs needs to take the complexity of wastewater composition into account. In addition, different from the EC approach, catalysts for PEC ammonia synthesis require excellent light absorption and photovoltaic conversion ability, which require a suitable bandgap structure and a rationally designed surface of the catalyst.

**TABLE 1:** Summary of the recent EC ammonia synthesis results based on TM‐based catalysts.

EC‐Catalysts	Electrolyte	NH_3_ yield rate	FE	Potential	Ref.
MV‐MoN@NC	0.1 M HCl_‐NRR_	76.9 µg h^−1^ mg^−1^_cat_	6.9%	−0.2 V_RHE_	[36]
Mo_SA_/CMF‐S	0.1 M HCl_‐NRR_	46.6 µg h^−1^ mg^−1^_cat_	28.9%	−0.2 V_RHE_	[37]
MoS_2_	0.1 M K_2_SO_4‐NRR_	66.74 µg h**^−^** ^1^ mg**^−^** ^1^	14.68%	−0.6 V_RHE_	[39]
Mo_2_C/C	0.5 M Li_2_SO_4‐NRR_	11.3 µg h^−1^ mg^−1^	7.8%	−0.3 V_RHE_	[44]
MoSAs‐Mo_2_C	0.1 M K_2_SO_4‐NRR_	16.1 µg h^−1^ cm^−2^_cat_	7.1%	−0.25 V_RHE_	[45]
Mo/HNG	0.05 M H_2_SO_4‐NRR_	3.6 µg h^−1^ mg^−1^_cat_	50.2%	−0.05 V_RHE_	[47]
CeO_2_‐MoN	0.1 M Na_2_SO_4‐NRR_	27.5 µg h^−1^ mg^−1^	17.2%	−0.3 V_RHE_	[50]
MoS_2_/C_3_N_4_	0.1 M Na_2_SO_4‐NRR_	18.5 µg h^−1^ mg^−1^	17.8%	−0.3 V_RHE_	[110]
MnMoO_4_	0.5 M LiClO_4‐NRR_	60.3 µg h^−1^ mg^−1^	14.7%	−0.4 V_RHE_	[111]
*α*‐MoB	0.1 M HCl_‐NRR_	5.45 µg h^−1^ mg^−1^	17.17%	−0.15 V_RHE_	[112]
Fe‐Fe_3_O_4_	0.1 M Na_2_SO_4‐NRR_	24.6 µg h^−1^ mg^−1^_cat_	53.2%	−0.4 V_RHE_	[52]
FeOOH	0.1 M LiClO_4‐NRR_	27.3 µg h^−1^ mg^−1^_cat_	14.6%	−0.4 V_RHE_	[53]
FeS_2_	0.1 M Na_2_SO_4‐NRR_	37.2 µg h^−1^ mg^−1^_cat_	11.2%	−0.5 V_RHE_	[55]
Fe‐MoS_2_	0.1 M KCl_‐NRR_	97.5 µg h^−1^ cm^−2^	31.6%	−0.2 V_RHE_	[56]
Fe_SAC_SC	0.1 M KOH_‐NRR_	8.8 µg h^−1^ mg^−1^	6.1%	−0.1 V_RHE_	[58]
FeMoPPc	0.1 M KOH_‐NRR_	36.33 µg h^−1^ mg^−1^_cat_	20.62%	−0.3 V_RHE_	[59]
F‐Fe@F‐G	1 M KOH_‐NRR_	53.3 µg h^−1^ mg^−1^	41.6%	−0.38 V_RHE_	[60]
Fe–N/C	0.1 M KOH_‐NRR_	34.83 µg h^−1^ mg^−1^_cat_	9.28%	−0.2 V_RHE_	[113]
Fe_3_C/Fe_3_O_4_	0.1 M HCl_‐NRR_	25.7 µg h^−1^ mg^−1^_cat_	22.5%	−0.2 V_RHE_	[114]
Fe−TiO_2_	0.5 M H_2_SO_4‐NRR_	12.9 mmol h^−1^ g^−1^_cat_	8.85%	3.5 V	[115]
Li‐TiO_2_	0.5 M LiClO_4‐NRR_	8.7 µg h^−1^ mg^−1^_cat_	18.2%	−0.4 V_RHE_	[61]
V‐TiO_2_	0.5 M LiClO_4‐NRR_	17.73 µg h^−1^ mg^−1^_cat_	15.3%	−0.5 V_RHE_	[62]
Mn‐TiO_2_	0.1 M Na_2_SO_4‐NRR_	20.05 µg h^−1^ mg^−1^_cat_	11.93%	−0.5 V_RHE_	[63]
TiO_2_/CeO_2_	0.1 M HCl_‐NRR_	8.8 µg h^−1^ mg^−1^_cat_	6.8%	−0.25 V_RHE_	[116]
MnO_2_	0.1 M Na_2_SO_4‐NRR_	147.2 µg h^−1^ mg^−1^_cat_	11%	−0.75 V_RHE_	[64]
BiO*_x_*	0.5 M K_2_SO_4‐NRR_	113 µg h^−1^ mg^−1^_cat_	30%	−0.8 V_RHE_	[117]
Cu foam	0.25 M Li_2_SO_4‐NORR_	517.1 µmol h^−1^ cm^−2^	93.5%	−0.9 V_RHE_	[67]
Cu	0.1 M NaOH_‐NORR_	1806 µmol h^−1^ cm^−2^	100%	0 V_RHE_	[118]
Cu@Cu/C	0.05 M H_2_SO_4‐NORR_	1180.5 µg h^−1^ cm^−2^	93%	−0.1 V_RHE_	[69]
Cu@Co	0.1 M Na_2_SO_4‐NORR_	627.20 µg h^−1^ cm^−2^	76.54%	−0.5 V_RHE_	[119]
MoS_2_/GF	0.1 M HCl_‐NORR_	99.6 µmol h^−1^ cm^−2^	76.6%	−0.7 V_RHE_	[70]
MoC/NCS	0.1 M HCl_‐NORR_	1350 µg h^−1^ cm^−2^	89%	−0.8 V_RHE_	[71]
NiNC@CF	PBS_‐NORR_	94 µmol h^−1^ cm^−2^	87%	−0.5 V_RHE_	[72]
Ni‐NCNR	0.1 M HCl_‐NORR_	23.8 µmol h^−1^ cm^−2^	85.5%	0.1 V_RHE_	[73]
Fe_2_O_3_	0.1 M Na_2_SO_4‐NORR_	78.02 µmol h^−1^ cm^−2^	86.73%	−0.4 V_RHE_	[74]
NiFe‐LDH	0.25 M Li_2_SO_4‐NORR_	112 µmol h^−1^ cm^−2^	82%	−0.7 V_RHE_	[75]
Cu‐PTCDA	0.1 M PBS_‐NtrRR_	436 µg h^−1^ cm^−2^	77%	−0.4 V_RHE_	[78]
Cu (1 1 1)	1 M KOH_‐NtrRR_	390.1 µg h^−1^ mg^−1^	99.7%	−0.15 V_RHE_	[120]
Cu/Cu_2_O	0.5 M Na_2_SO_4‐NtrRR_	0.2449 mmol h^−1^ cm^−2^m^−2^	95.8%	−0.85 V_RHE_	[79]
CoP‐CNS	1 M OH^−^_‐NtrRR_	8.47 mmol h^−1^ cm^−2^	88.6%	−1.03 V_RHE_	[81]
CoO*_x_*	0.1 M KOH_‐NtrRR_	82.4 mg h^−1^ mg^−1^_cat_	93.4%	−0.3 V_RHE_	[121]
CoP PANSs	0.5 M K_2_SO_4‐NtrRR_	19.28 mg h^−1^ mg^−1^_cat_	94.24%	−0.5 V_RHE_	[122]
Fe SAC	0.1 M K_2_SO_4‐NtrRR_	0.46 mmol h^−1^ cm^−2^	75%	−0.85 V_RHE_	[82]
Fe/Cu‐HNG	1 M KOH_‐NtrRR_	1.08 mmol h^−1^ mg^−1^	92.51%	−0.5 V_RHE_	[83]
Fe_3_C/NC	1 M KOH_‐NtrRR_	1.19 mmol h^−1^ mg^−1^	96.7%	−0.5 V_RHE_	[123]
Fe_3_O_4_/SS	0.1 M NaOH_‐NtrRR_	10.1 mg h^−1^ cm^−2^	91.5%	−0.5 V_RHE_	[124]
TiO_2 − _ *_x_*	0.5 M Na_2_SO_4‐NtrRR_	0.045 mmol h^−1^ mg^−1^	85%	−1.6 V_SCE_	[125]
In‐S‐G	1 M KOH_‐NtrRR_	220 mmol h^−1^ g^−1^_cat_	75%	−0.5 V_RHE_	[126]

**TABLE 2:** Summary of the recent PEC ammonia synthesis results based on TM‐based catalysts.

PEC‐Catalysts	Electrolyte	NH_3_ yield rate	FE	Potential	Ref.
MoS_2_@TiO_2_	0.1 M Na_2_SO_4‐NRR_	1.42 µmol h^−1^ cm^−2^	65.52%	−0.2 V_RHE_	[87]
Mo_2_C/C	0.1 M Li_2_SO_4‐NRR_	6.6 µg h^−1^ mg^−1^	37.2%	0.2 V_Ag/AgCl_	[88]
MoSe_2_@g‐C_3_N_4_	0.1 M KOH_‐NRR_	7.72 µmol h^−1^ cm^−2^	28.91%	−0.3 V_RHE_	[89]
Mo‐WO_3_@CdS	0.1 M Li_2_SO_4‐NRR_	38.99 µg h^−1^ mg^−1^_cat_	36.72%	−0.3 V_RHE_	[91]
CoPi/Ti‐Fe_2_O_3_	0.2 M NaOH_‐NRR_	1021.5 µg h^−1^ mg_Co_^−1^	11.9%	1.2 V_RHE_	[93]
Cu_2_O	0.1 M KOH_‐NRR_	7.2 µg h^−1^ cm^−2^	20%	0.4 V_RHE_	[94]
BDB NR	0.05 M H_2_SO_4‐NRR_	29.2 mg_NH3_ h^−1^ g^−1^_cat_	8.3%	0.48 V_RHE_	[95]
R‐BiOI	0.5 M Na_2_SO_4‐NRR_	1.4 mmol h^−1^ m^−2^	N/A	0.4 V_RHE_	[96]
Cu‐MOF/Cu_2_O	0.1 M Li_2_SO_4‐NRR_	7.16 mmol h^−1^ m^−2^	N/A	0.5 V_RHE_	[127]
p‐BiVO_4_	0.1 M Li_2_SO_4‐NRR_	0.116 µmol h^−1^ cm^−2^	16.2%	−0.1 V_RHE_	[128]
Ni@NC	0.1 M HCl_‐NORR_	22 µmol h^−1^ cm^−2^	>50%	2.1 V	[97]
CuCSi	1 M KOH_‐NtrRR_	115.3 µmol h^−1^ cm^−2^	88.8%	−0.6 V_RHE_	[102]
ZnIn_2_S_4_/BiVO_4_	0.5 M Na_2_SO_4‐NtrRR_	29.95 µg h^−1^ cm^−2^	37.2%	−0.1 V_RHE_	[103]
CoFeMnO/BiVO_4_	0.5 M Na_2_SO_4‐NtrRR_	17.82 µg h^−1^ cm^−2^	32.8%	−0.1 V_RHE_	[104]
TiO*_x_*/CdS/CZTS	0.1 M K_2_SO_4‐NtrRR_	8.21 µmol h^−1^ cm^−2^	89.1%	−0.2 V_RHE_	[107]
Si@TiO_2_	1 M KNO_3‐NtrRR_	1074 µg_NH3_ h^−1^ cm^−2^	94.3%	−0.6 V_RHE_	[108]
OD‐Co	1 M KNO_3‐NtrRR_	N/A	92.37%	−0.8 V_RHE_	[109]
Co_0.95_Ni_0.05_/Si	0.25 M K_2_B_4_O_7‐NtrRR_	2054 µg h^−1^ cm^−2^	98.6%	−0.1 V_RHE_	[129]
CeO_2_–C/BiVO_4_	0.5 M Na_2_SO_4‐NtrRR_	21.81 µg h^−1^ cm^−2^	32.2%	−0.1 V_RHE_	[130]
CuPc/CeO_2_	0.1 M PBS_‐NtrRR_	1.16 µmol h^−1^ m^−2^	33%	−0.6 V_RHE_	[131]

## 3 Advanced TMCS for NH_3_ Decomposition

The ammonia decomposition reaction has triggered great attention as a promising strategy for carbon‐free hydrogen generation owing to its excellent volumetric energy density (12.7 MJ L^−1^) and high hydrogen content (17.6 wt%) [132, 133]. Up to now, a variety of traditional techniques, including biological conversion [134, 135], membrane separation [136, 137], ion exchange [138, 139], and breakpoint chlorination [140, 141] have been explored for ammonia decomposition. However, traditional NH_3_ decomposition approaches still suffer from high costs and poor removal efficiency [142, 143]. In contrast, EC and PEC NH_3_ dissociation are regarded as promising alternate technologies because of their high conversion efficiency, cost‐effectiveness, and environmental friendliness [144]. Ideally, these technologies can convert ammonia into non‐toxic N_2_ and H_2_O molecules, simultaneously coupled with the generation of clean fuel H_2_. However, in the processes of catalytic reactions, the reaction mechanisms of different catalysts will lead to different ammonia oxidation reaction (AOR) products. Thus, an in‐depth understanding of the properties of reactants, intermediates, and the final products is of great significance in guiding the rational design of high‐performance AOR catalysts at the molecular or atomic level.

**FIGURE 11:**
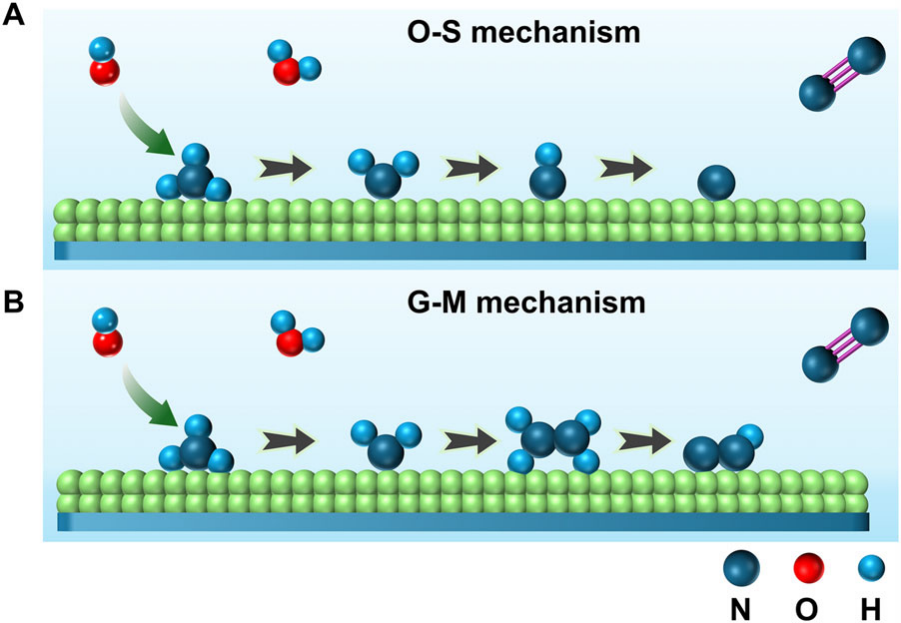
The mechanisms of ammonia oxidation reaction. (A) Oswin and Salomon (O‐S) and (B) Gerischer and Maurer (G‐M). Reproduced with permission [151]. Copyright 2023, Wiley‐VCH.

The typical AOR in aqueous conditions is a multi‐proton‐coupled‐electron‐transfer process, making it more complex than four‐electron‐coupled OER [145]. Considering the competition between OER and the AOR at the anode part, the catalyst needs to have excellent adsorption selectivity of ammonia. In acidic conditions, AOR pathway could turn to NH_4_^+^ formation, resulting in indirect oxidation and poor AOR efficiency due to the surface repulsion between NH_4_^+^ and the catalyst [146]. Ammonia can effectively maintain its molecular form in alkaline aqueous electrolytes, making the AOR more favorable in alkaline conditions [147]. Therefore, alkaline conditions are predominantly used in electrolyte environments [148]. Typically, electrocatalytic AOR is considered to follow two pathways, as illustrated in Figure 11. As shown in Figure 11A, proposed by Oswin and Salomon in 1963, NH_3_ molecules tend to be adsorbed on the active sites and undergo multiple dehydrogenations to obtain the adsorbed atomic nitrogen (N_ads_) intermediate, followed by the subsequent coupling of the two N_ads_ to form nitrogen molecules, which is noted as the O‐S mechanism. It should be noted that the dehydrogenation of NH_2_(ads) to NH(ads) is a rate‐determining step for O‐S mechanism at low current density; while the dimerization of N_ads_ to N_2_ molecules is considered as the rate‐determining step at high current density [149]. Notably, the N_ads_ desorption efficiency is also crucial, while the delayed desorption could lead to the blocking of the catalytic sites, which is called catalyst poisoning [150]. In a subsequent development, Gerischer and Maurer proposed another G‐M mechanism [151, 152]. Differently, the G‐M mechanism indicates that substances partially dehydrogenated by ammonia will dimerize into hydrazine‐like species *N_2_H*_x_* (*x* = 2, 3, 4) and finally dehydrogenate to N_2_ molecules production, without the N_ads_ formation process, as illustrated in Figure 11B. In this mechanism, the NH*_x_* (ads) dimerization process is additionally pointed out as the rate‐determining step. However, for both AOR mechanisms, the poor reaction kinetics of AOR usually result in high overpotential and poor stability issues. In most cases, ammonia can be oxidized through different reaction pathways toward various nitrogen compounds such as nitrogen, nitrate ion, and NO*_x_* during electro‐oxidation [153]. Therefore, the development of suitable catalysts with appropriate reactants, excellent intermediate adsorption and desorption properties are considered to be the crucial point [154].

Traditional precious metals such as Pt, Pd, Ru, and Ag‐based catalysts exhibit excellent low‐temperature activity toward NH_3_ oxidation reaction [155]. However, it is non‐negligible that their widespread applications are significantly hindered by the issues of high cost and relatively poor selectivity toward N_2_ [156, 157]. Therefore, much research on NH_3_‐to‐H_2_ conversion has focused on non‐noble TMC‐based catalysts [158]. Moreover, the EC AOR still suffers from its sluggish catalytic kinetics, which requires high overpotential to activate the efficient ammonia conversion for H_2_ generation. In contrast, the PEC process can significantly reduce the overpotential owing to the photovoltage stimulated by solar‐induced charge carriers, which is also beneficial for efficient long‐term ammonia degradation [141]. Although the research on EC/PEC ammonia oxidation to hydrogen generation is still in its start‐up stage, considering its vast potential in the future “green‐hydrogen” economy development, we summarize the existing relevant studies in the following section.

### 3.1 TMCs for EC NH_3_ Decomposition

**FIGURE 12:**
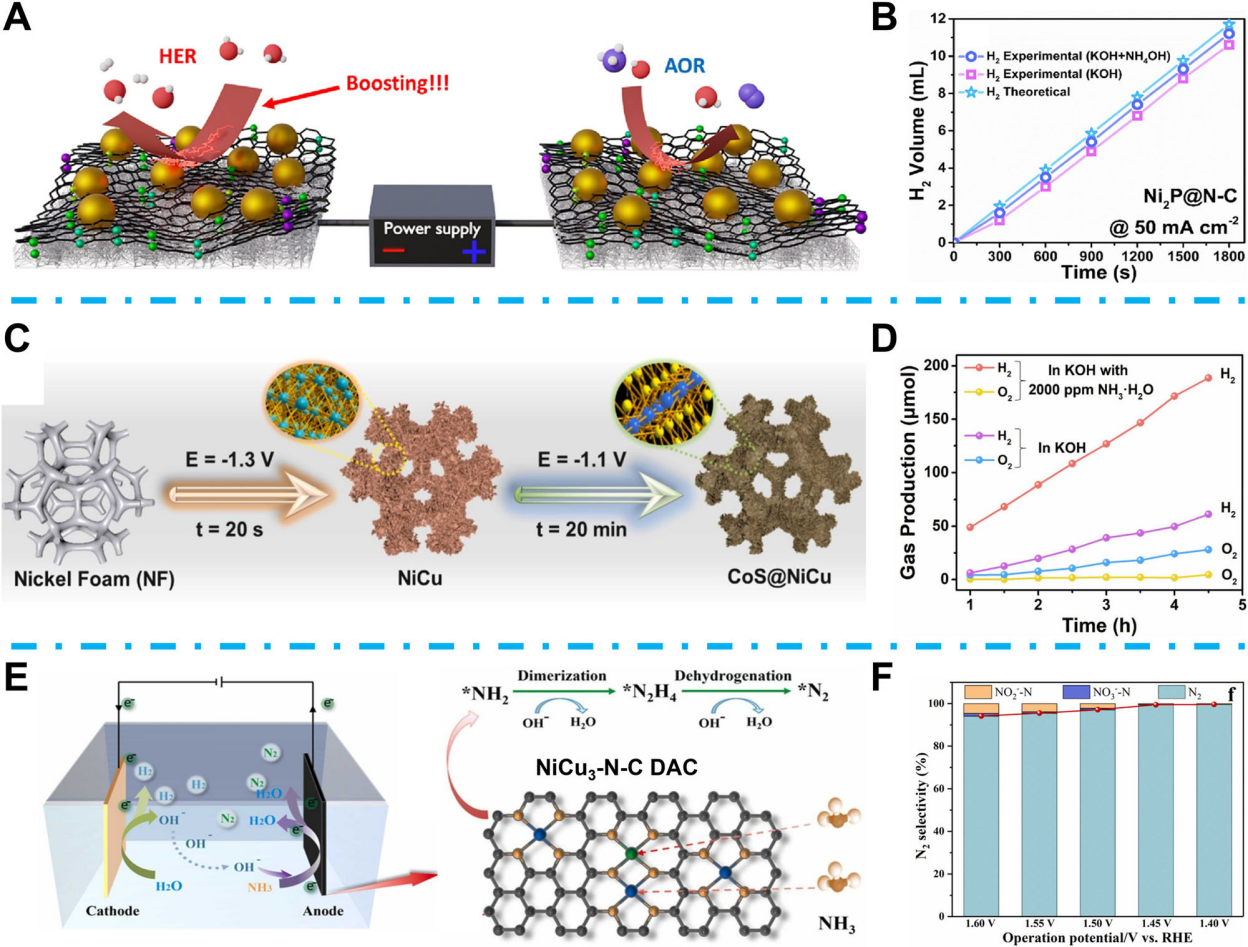
(A) Schematic illustration of the AOR and HER reaction mechanism based on the Ni_2_P@N‐C. Reproduced with permission [163]. (B) H_2_ generation volume of Ni_2_P@N‐C. Reproduced with permission [163]. Copyright 2023, Elsevier. (C) Schematic illustration of CoS@NiCu synthesis process. Reproduced with permission [164]. (D) Gas production rates of CoS@NiCu in the OER‐HER and AOR‐HER systems. Reproduced with permission [164]. Copyright 2023, Elsevier. (E) Schematic illustration of NiCu_3_‐D‐C DAC catalysts for PEC AOR. Reproduced with permission [167]. (F) N_2_ selectivity of NiCu_3_‐D‐C DAC at different potentials. Reproduced with permission [167]. Copyright 2023, Elsevier.

EC AOR is an eco‐friendly technology that can be effectively controlled using electrical input [15]. So far, various TMCs for EC ammonia oxidation have been investigated. As a typical transition metal, Nickel shows enormous potential in ammonia fuel cells owing to its excellent ammonia oxidation catalytic property and hydrogen oxidation efficiency resulting from its outstanding electrical conductivity and electrocatalytic activity [159]. However, the coarsening and agglomeration of Ni during the AOR process and undesired nitrogen adsorption enthalpy ($\Delta \;H_{\mathrm{Ni}-N}=-240\;\mathrm{kj}\;\mathrm{mo}l^{-1}$) restricted its EC AOR efficiency. Huang et al. used pure metallic nickel foam as the anode for ammonia oxidation and confirmed that the in situ formed surface NiOOH triggered the ammonia oxidation to nitrogen [160]. Wang et al. proposed that the wide d‐band of pure nickel could result in poor NH_3_ adsorption properties, which makes strengthening the binding strength a critical strategy for nickel‐based catalysts on EC AOR [161]. Myers et al. proposed that introducing phosphorus could efficiently optimize the NH_3_ adsorption property, which resulted from the increased d band by the interaction of Ni and P elements [162]. In light of this, Sim's group fabricated Ni_2_P nanoparticles onto a nitrogen‐doped‐carbon substrate, and the formed Ni_2_P@NC was applied to an ammonia electrolysis cell (Figure 12A) [163]. The Ni‐P demonstrated enhanced corrosion resistance and better stability. However, the electrical conductivity of metal phosphide is still unsatisfactory compared with that of pure metal. In this research, the introduced N‐doped C substrate can enhance electrical conductivity and catalytic kinetics, achieving a high anodic current density of 47.4 mA cm^−2^ at 1.6 V_RHE_, 2.9 times more than bare Ni_2_P. Consequently, Ni_2_P@NC confirmed a high ammonia decomposition rate of 78%. At the same time, the H_2_ generation through AOR within 30 min reached 11.2 mL, higher than that of HER (Figure 12B). In addition, the efficient utilization of the lone pairs in ammonia remains a big challenge for the exploration of highly active AOR catalysts. Lin et al. indicated that the strong electron pair interaction between the transition metal sulfide and ammonia can effectively boost the AOR activity. In their research, they proposed a 3D CoS@NiCu catalyst, self‐supported on Ni foam (Figure 12C). They demonstrated that the extended Co─S bond resulting from the adsorbed NH_3_ molecule could lead to accelerated CoOOH active sites formation for AOR. Finally, the CoS@NiCu showed an optimized energy barrier and promoted catalytic kinetics for AOR. The assembled CoS@NiCu/CoS@NiCu AOR‐HER system showed an excellent H_2_ yield rate of 41.9 µmol h^−1^, 3.2 fold higher than the OER‐HER system (Figure 12D) [164]. Downsizing metal particles to single‐metal atoms is an efficient way to increase active site utilization efficiency, and the resulting single‐atom catalysts (SACs) have attracted significant research due to their combined features of fully exposed active sites and recyclable properties [165]. Researchers have found that, unlike SAC, a catalyst with a diatomic structure can provide superior catalytic performance compared to SAC owing to cooperation between the two atoms [166]. Wu et al. proposed a diatomic site catalyst (DAC) for highly efficient electrocatalytic AOR [167]. In their work, Ni and Cu bimetallic sites were anchored on nitrogen‐doped carbon (N–C) with a three‐dimensional porous skeleton morphology using a solid‐phase pyrolysis method. At the same time, the N–C substrate provided an excellent anchor site for immobilizing the active metal center (Figure 12E). The coexisting Cu‐N_4_ and Ni‐N_4_ sites were favorable for the internal electronic structure optimization, and nitrogen intermediates adsorption for EC AOR, which further accelerated the proto‐electron transfer efficiency. As a result, this NiCu_3_‐N‐C DAC showed significant EC AOR to N_2_ selectivity (>95%) with the applied bias from 1.4 to 1.6 V_RHE_ (Figure 12F).

**FIGURE 13:**
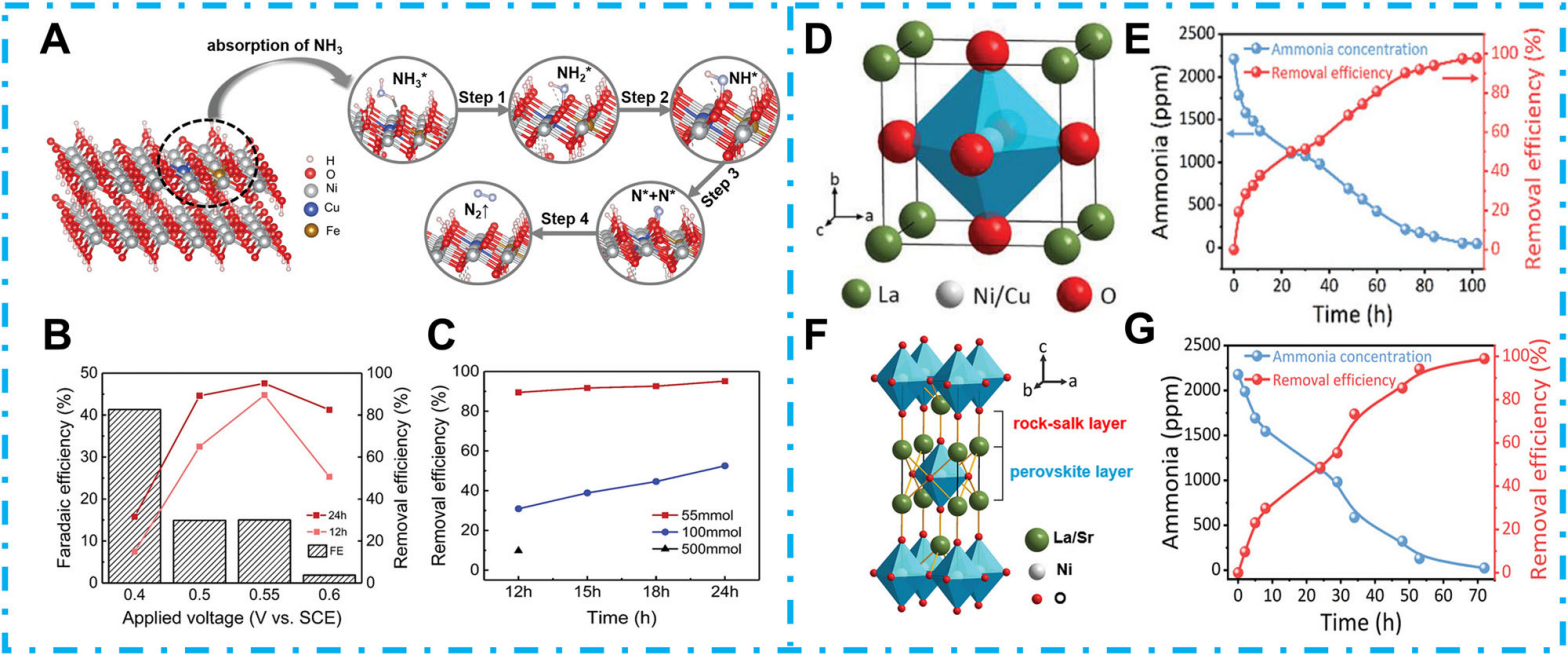
(A) Schematic illustration of Ni‐Cu‐Fe‐OOH ammonia oxidation process. Reproduced with permission [169]. (B) Ammonia removal efficiency and FE of the activated NiCuFe electrode. Reproduced with permission [169]. (C) Ammonia decomposition efficiency of NiCuFe under different NH_3_ concentrations. Reproduced with permission [169]. Copyright 2021, Wiley‐VCH. (D) Schematic diagram of LaNi_0.5_Cu_0.5_O_3−_ *_δ_* perovskite structure. Reproduced with permission [170]. (E) Ammonia decomposition efficiency of SAE‐LNCO55‐Ar at 1.23 V. Reproduced with permission [170]. Copyright 2021, Wiley‐VCH. (F) Crystal structure illustration of the RP‐type oxide. (G) Ammonia decomposition efficiency of SAE‐LSNC‐Ar at 1.22 V. Reproduced with permission [172]. Copyright 2022, Wiley‐VCH.

Heterogeneous element doping is considered an efficient strategy to enhance the catalytic activity and metal‐based catalyst stability. Xu et al. found that NiCu bimetal on carbon paper exhibited higher catalytic activity than Ni or Cu, and the ammonia removal efficiency can achieve ≈80% by using NiCu anode [168]. Zhu et al. proposed that although different Ni‐based materials have been applied to EC AOR, the formed NiOOH during EC activation would be the active site for AOR. However, they found that the weak electrostatic interaction between adsorbed NH_3_ and O_Ni‐O_ would lead to sluggish AOR kinetics because of the high energy barrier. Cu doping can enhance the electron density for Ni_(1–_ *_x_*_)_Cu*_x_* OOH, which is beneficial for the NH_3_ adsorption and improves AOR performance. However, the increased Cu loading amount would lead to decreased AOR property. To address this issue, Zhu et al. introduced Fe^3+^ into Ni_(1–_ *_x_*_)_Cu*_x_* OOH due to the suitable radius and lower electronegativity of Fe (Figure 13A) [169]. The co‐existed Cu–Fe dopants led to a modulated surface electron distribution, which helped the Ni‐Cu‐Fe‐OOH achieve a high ammonia consumption rate of 89.4% for 12 h (95.1% for 24 h) at 0.55 *V*_SCE_ (Figure 13B). Moreover, the Ni‐Cu‐Fe‐OOH also demonstrated excellent AOR property in high‐concentration ammonia solution, confirming 55% consumption rate for 24 h AOR in 100 mM ammonia electrolyte (Figure 13C). Zhang et al. proposed that perovskite with the structure of ABO_3_ can be applied for AOR [170]. They applied Cu and Ni to occupy the B site of the BO_6_ octahedron (Figure 13D) to fabricate LaNi_0.5_Cu_0.5_O_3‐δ_ perovskite, which can regulate the B site oxidation states and enhance the surface defect states. This LaNi_0.5_Cu_0.5_O_3‐δ_ demonstrated excellent AOR and HER properties, while the assembled SAE‐LNCO55‐Ar finally achieved 100% ammonia decomposition after 100 h AOR (Figure 13E) at 1.23 V. Feng et al. proposed a rechargeable Zn–NH_3_ battery by using a bifunctional Mo_2_C/NiCu@C catalyst, which achieved efficient H_2_ evolution. The hierarchical carbon‐coated Mo_2_C@C performs as HER active sites, while NiCu@C dotted on/in carbon spheres serves as AOR active sites. The AOR to N_2_ generation was conducted during cathodic charging, while the water reduction to H_2_ generation was carried out during cathodic discharging. Finally, this rechargeable Zn–NH_3_ battery achieved an efficient NH_3_‐to‐H_2_ conversion with a high FE of 91.6% and excellent long‐term stability for more than 900 cycles at 20 mA cm^2^ [171]. To further enhance the AOR property, Zhang et al. substituted the A site of ABO_3_ perovskite to further improve the surface oxygen vacancies. They chose low‐valenced Sr for an A‐site substitution. They found that 75% Sr‐doped La_0.5_Sr_1.5_Ni_0.9_Cu_0.1_O_4−_ *_δ_* showed favorable oxygen vacancies (Figure 13F). Finally, the assembled SAE‐LSNC‐Ar cell achieved 100% ammonia decomposition after 72 h AOR (Figure 13G) at 1.22 V, and achieved 95% ammonia decomposition for 170 h AOR in real wastewater [172].

### 3.2 TMCs for PEC NH_3_ Decomposition

**FIGURE 14:**
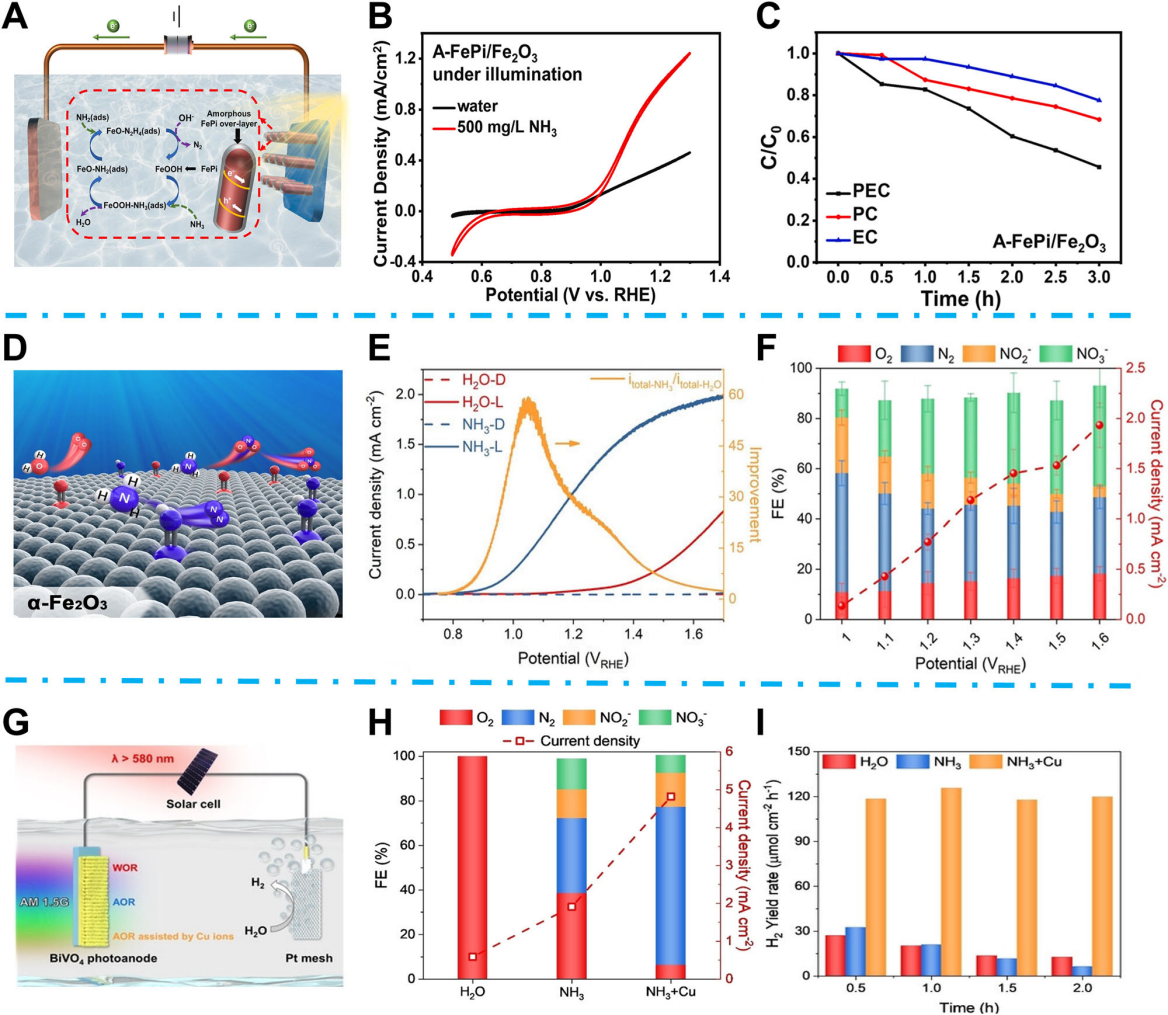
(A) Schematic illustration of the PEC AOR system based on FePi/Fe_2_O_3_ photoanode. (B) PEC AOR and OER CV curves compare FePi/Fe_2_O_3_ under 1 sun illumination. (C) Ammonia degradation rate of FePi/Fe_2_O_3_ through different testing conditions at 1.23 V_RHE_ under 1 sun illumination. Reproduced with permission [14]. Copyright 2020, Elsevier. (D) Schematic illustration of α‐Fe_2_O_3_ photoanode for PEC AOR [178]. (E) LSV curves of α‐Fe_2_O_3_ photoanode for AOR and OER under 1 sun illumination [178]. (F) The PEC AOR and OER FE of α‐Fe_2_O_3_ photoanode. Reproduced with permission [178]. Copyright 2022, Wiley‐VCH. (G) Schematic illustration of PEC AOR for a hydrogen production device based on BiVO_4_ photoanode [179]. (H) The PEC current density and FE for different products of BiVO_4_ photoanode [179]. (I) H_2_ yield rate of Cu‐assisted AOR on BiVO_4_. Reproduced with permission [179]. Copyright 2023, Wiley‐VCH.

In recent years, inspired by PEC OER, PEC AOR has attracted increased interest as it is considered an efficient approach to overcoming sluggish catalytic kinetics [173, 174]. Typically, the indirect radical‐mediated strategy was applied for the solar‐assisted AOR process, while the ammonia was oxidized by the in situ formed oxysulfur radicals or chlorine species, which resulted in poor selectivity and enhanced cost issues [175, 176]. Zhang et al. designed a BiVO_4_/WO_3_ heterojunction, while the fine‐tuned band edge position regulated the selective oxidation of chlorine species to Cl**·**for efficient PEC ammonia decomposition [177]. Despite these oxidant mediators can enhance the efficiency of the AOR by facilitating charge transfer processes and improving the kinetics of the reaction, they also pose several challenges in terms of costs, current efficiency (Faradaic efficiency, FE), and toxic by‐products. In contrast, the direct PEC AOR without additional redox mediators has shown its advantages in recent years. Our group designed a highly activated ferric phosphate amorphous layer covered with hematite photoanode (FePi/Fe_2_O_3_) for direct PEC AOR (Figure 14A). The surface FePi layer can be converted to FeOOH, which further enhances the active sites for ammonia adsorption and accelerates the PEC AOR kinetics [14]. As a result, the activated FePi/Fe_2_O_3_ (A‐FePi/Fe_2_O_3_) showed significantly enhanced PEC AOR photocurrent response (Figure 14B). During 3 h PEC AOR with 500 ppm ammonia under one sun illumination, A‐FePi/Fe_2_O_3_ achieved 54.4% ammonia decomposition, which was much higher compared to that of PC AOR (32%) and EC AOR (22%), as shown in Figure 14C. Our research confirmed the feasibility of highly efficient PEC AOR in chlorine‐free conditions. In light of this, Wu et al. fabricated α‐Fe_2_O_3_ and investigated its PEC AOR property in near‐neutral conditions (Figure 14D) [178]. In their research, the PEC AOR photocurrent density in the electrolyte with different ammonia concentrations was investigated; they found that the onset potential of α‐Fe_2_O_3_ was significantly reduced for 300 mV, while the photocurrent of AOR enhanced to 10 to 55 times compared to that of PEC OER at 1.0 to 1.4 V_RHE_ (Figure 14E), and the current density was enhanced with the increase of ammonia concentration. Their research indicated that the enhanced PEC AOR property can be assigned to the non‐radical nucleophilic attack from ammonia to surface Fe–O and Fe–N species, which led to nitrate and nitrogen formation. Finally, the α‐Fe_2_O_3_ achieved a high PEC AOR FE of 81.2% while the competitive PEC OER was just 10.8% at 1.1 V_RHE_ (Figure 14F). Compared to α‐Fe_2_O_3_, BiVO_4_ shows better PEC energy conversion property owing to its favorable hole mobility of 2 cm^2^ V^−1^ s^−1^ (α‐Fe_2_O_3_: ≈10^−7^–10^−4^ cm^2^ V^−1^ s^−1^). However, the photostability of BiVO_4_ seriously restricts its application in the PEC system. Different strategies have been applied for the harmful photo‐corrosion in PEC OER, but considering the competition between AOR and OER, a new approach is required to enhance the PEC AOR stability for BiVO_4_ [145]. Zhao et al. introduced Cu ions into the electrolyte of the PEC AOR system (Figure 14G), which significantly enhanced the PEC AOR stability of the BiVO_4_ photoanode [179]. They found that the introduced Cu ion could form a Cu‐NH_3_ complex and adsorbed on the surface of BiVO_4_, which is beneficial for the surface defect passivation and further suppresses the OER. The corresponding AOR photocurrent of BiVO_4_ in Cu introduced electrolyte showed more than two times enhancement at 1.23 V_RHE_. Furthermore, the PEC AOR FE of BiVO_4_ in Cu‐NH_3_ electrolyte was significantly enhanced to 93.8%. In comparison, the OER FE was suppressed to 6.2% (Figure 14H). The introduced Cu coordination led to an activated NH_3_ molecule, which accelerated the charge transfer of AOR, resulting in significantly enhanced PEC AOR catalytic activity and promoted stability of BiVO_4_. Consequently, the corresponding assembled PEC HER cell based on BiVO_4_ photoanode and Pt mesh and Cu induced electrolyte achieved seven times enhancement on H_2_ evolution property, reaching 120 µmol cm^−2^ h^−1^ (Figure 14I).

## 4 Outlook

In general, EC and PEC ammonia conversion processes have shown significant potential for carbon‐free energy development, while exploring catalysts with low cost, high catalytic activity, and excellent stability is the key to achieving highly efficient, practical nitrogen‐based energy innovation. Transition metal‐based materials have been widely explored as catalysts for EC/PEC ammonia synthesis and oxidation to hydrogen generation, while significant achievements have been promoted. Nevertheless, from the perspective of large‐scale commercial applications, there remains a long way to go. Considering the competition of ammonia catalytic conversion with water splitting in an aqueous environment, the reactant adsorption selectivity would be the crucial point of the catalyst exploration for both ammonia synthesis and ammonia oxidation properties. However, given multiple EC/PEC ammonia synthesis pathways (NRR, NORR, NtrRR) and the complicated reaction mechanism of ammonia synthesis and oxidation to the hydrogen generation process, TMCs must be explored for corresponding reaction conditions. In particular, for NRR, the catalysts research should meet the high cracking bonding energy requirement of N≡N. Compared to NRR, NORR, and NtrRR are more thermodynamically favorable owing to the lower bonding energy of N═O, but 5e^−^ conversion of NORR poses a challenge to the efficient utilization of electrons. At the same time, the complicated reaction pathways and intermediates of NtrRR lead to new requirements for highly efficient NH_3_ production. The highly selective AOR to N_2_ pathway and efficient product desorption property are crucial for ammonia decomposition to achieve efficient NH_3_ to H_2_ conversion. Compared to the EC catalytic process, the PEC approach demonstrated great potential for highly efficient ammonia cycling, which is beneficial for using clean and sustainable solar energy, providing promoted catalytic kinetics (Table 3). Therefore, the catalyst exploration for PEC ammonia synthesis and decomposition to H_2_ generation requires considering excellent light absorption, photoelectric conversion efficiency, and strategies to mitigate photocorrosion, which is a crucial issue for photoelectrodes in the N‐cycling system.

Considering that the reactants adsorption, product desorption, and light absorption in the PEC catalysis system are closely related to the surface property of the catalysts, surface engineering will be a promising strategy in the future ammonia cycling catalysts exploration, apart from selecting suitable catalysts. Furthermore, the exploration of catalysts should not be limited to ideal laboratory conditions. More efforts are required for practical applications. For reactions involving gaseous reactants (NRR, NORR), the catalyst exploration should take the low gas solubility into account, while for the liquid reactants (NtrRR AOR), the complex components in the actual sewage should be considered to develop efficient and highly stable catalysts.

**TABLE 3:** Summary of the recent EC and PEC ammonia oxidation results based on TM‐based catalysts.

Catalysts	Ammonia concentration	AOR rate	HER rate	Potential	Ref.
Ni_2_P@N‐C	0.5 M_‐EC_	77.78%	2.1 mmol h^−1^ cm^−2^	1.6 V_RHE‐8 h_	[163]
CoS@NiCu	2000 ppm_‐EC_	N/A	41.9 µmol h^−1^	1.6 V	[164]
NiCu_3_‐NC	700 mg L^−1^_‐EC_	99.16%	N/A	1.5 V_RHE‐5 h_	[167]
NiCu	1100 ppm_‐EC_	80%	N/A	1.3 V_‐14 h_	[168]
NiCuFe	0.05 M_‐EC_	90%	N/A	0.55 V_SCE‐12 h_	[169]
LaNi_0.5_Cu_0.5_O_3‐_ *_δ_*	2210 ppm_‐EC_	100%	N/A	1.23 V_‐100 h_	[170]
Mo_2_C/NiCu@C	0.3 M_‐EC_	N/A	91.6%‐_FE_	0.48 V_RHE_	[171]
La_0.5_Sr_1.5_Ni_0.9_Cu_0.1_O_4–δ_	2177 ppm_‐EC_	95%	N/A	1.22 V_‐72 h_	[172]
CuO‐TiNTAs	500 mg L^−1^_‐PEC_	50.1%	19.6 µmol cm^−2^ h^−1^	1 V_‐2 h_	[173]
MoS_2_/WS_2_	20 mg L^−1^_‐PEC_	87%	N/A	0.6 V_Ag/AgCl‐6 h_	[176]
BiVO_4_/WO_3_	10 mg L^−1^_‐PEC_	99.3%	N/A	0 V_‐2 h_	[177]
FePi/Fe_2_O_3_	500 ppm_‐PEC_	54.4%	N/A	1.23 V_RHE‐3 h_	[14]
α‐Fe_2_O_3_	0.1 M_‐PEC_	81%_‐FE_	N/A	1 V_RHE‐2 h_	[178]
Cu/BiVO_4_	0.2 M_‐PEC_	93%_‐FE_	120 µmol cm^−2^ h^−1^	1.23 V_RHE_	[180]

## Conflicts of Interest

The authors declare no conflicts of interest.
